# Can speed be judged independent of direction?

**DOI:** 10.1167/18.6.15

**Published:** 2018-06-22

**Authors:** Catherine Manning, Rory Trevelyan Thomas, Oliver Braddick

**Affiliations:** catherine.manning@psy.ox.ac.uk; Department of Experimental Psychology, University of Oxford, Oxford, UK; Department of Experimental Psychology, University of Oxford, Oxford, UK; Department of Experimental Psychology, University of Oxford, Oxford, UK

**Keywords:** *speed discrimination*, *oblique effect*, *direction*, *bias*, *motion perception*

## Abstract

The ability to judge speed is a fundamental aspect of visual motion processing. Speed judgments are generally assumed to depend on signals in motion-sensitive, directionally selective, neurons in areas such as V1 and MT. Speed comparisons might therefore be expected to be most accurate when they use information within a common set of directionally tuned neurons. However, there does not appear to be any published evidence on how well speeds can be compared for movements in different directions. We tested speed discrimination judgments between pairs of random-dot stimuli presented side-by-side in a series of four experiments (*n* = 65). Participants judged which appeared faster of a reference stimulus moving along the cardinal or oblique axis and a comparison stimulus moving either in the same direction or in a different direction. The bias (point of subjective equality) and sensitivity (Weber fraction) were estimated from individual psychometric functions fitted for each condition. There was considerable between-participants variability in psychophysical estimates across conditions. Nonetheless, participants generally made more acute comparisons between stimuli moving in the same direction than those moving in different directions, at least for conditions with an upwards reference (∼20% difference in Weber fractions). We also showed evidence for an oblique effect in speed discrimination when comparing stimuli moving in the same direction, and a bias whereby oblique motion tended to be perceived as moving faster than cardinal motion. These results demonstrate interactions between speed and direction processing, thus informing our understanding of how they are represented in the brain.

## Introduction

**Figure 1:**
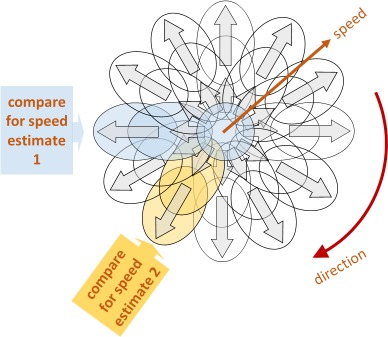
Schematic for a consensus model of the neural representation of motion. Each ellipse represents the velocity tuning of a particular neural channel. In this proposal, speed judgments are derived from the comparison of signals in channels with similar directional tuning but different speed tuning. Speed judgment of motion at −90° (“estimate 1”) therefore depends on a different set of neural channels from speed judgment of motion at −150° (“estimate 2”). Convention: 0° = vertically upwards motion.

Visual motion processing is one of the most extensively investigated aspects of vision, and one important aspect of this processing is the ability to judge and compare the speed of moving stimuli. It is generally supposed that our ability to judge motions is based on the pattern of activity within an array of directionally selective channels (Simoncelli & Heeger, 2001; Mather, 2011). Psychophysical and neuronal data indicate that the directional bandwidth of these channels is of the order of ±45° (Britten & Newsome, 1998; Fine, Anderson, Boynton, & Dobkins, 2004), so a considerable number are required to span the 360° range of possible directions. There are also a number of channels with distinct speed sensitivity, although there is some discrepancy between psychophysical data which suggest a small number of such channels, perhaps two or three (Thompson, 1984; Hess & Plant, 1985; Hess & Snowden, 1992) and data on speed selectivity of neurons in macaque area MT/V5, which indicate a more continuous range of peak speeds (Maunsell & van Essen, 1983; Mikami, Newsome, & Wurtz, 1986; Krekelberg & van Wezel, 2013). Figure 1 is a schema of this consensus model of the neural representation of motion.

On this “labelled line” scheme (Britten & Newsome, 1998; Krekelberg & van Wezel, 2013), when we make a judgment of speed, we are comparing the signals from two or more speed channels tuned to the same direction (Smith & Edgar, 1994; Hammett, Champion, Morland, & Thompson, 2005). If we are required to compare the speed of two different motions, then it might be expected that two speeds in the same direction would use information from a common set of direction channels, whereas two speeds in different directions would require a comparison across neural populations that are more separate. This postulate raises the question of whether such a comparison is more efficient or precise when channels with a common directional tuning are involved.

This question relates to a much broader, and rarely considered, issue in sensory science: Which neural operations allow us to compare representations carried by two different populations of neurons? Psychophysical tasks often require signals to be compared between neurons representing different parts of the visual field, and sometimes (as in the case suggested here) between neurons with different stimulus selectivity in dimensions other than the dimension directly under study. As a rare example of a study addressing this issue, Burbeck and Regan (1983) examined spatial frequency discrimination for gratings in the same or different orientations, and also orientation discrimination for the same or different spatial frequencies. These tasks are static analogues to our proposal for comparing speeds in the same or different directions. In each case, Burbeck and Regan reported that discrimination in one dimension was equally acute whether or not the stimuli were matched in the other, irrelevant, dimension. They concluded that “orientation and spatial frequency can be regarded as independent dimensions at the discrimination stage of visual processing” (p. 1693). This deduction poses the problem, but does not answer it, of how this “discrimination stage” operates on patterns of activation from neurons differently tuned in the other dimension—comparisons which can be almost arbitrarily set by the experimental design.

Although there are many published investigations of speed discrimination (for example Orban, de Wolf, & Maes, 1984; Smith & Edgar, 1994; McKee, Silverman, & Nakayama, 1986; Smith, 1987; De Bruyn & Orban, 1988; Henning & Derrington, 1994; Verghese & Stone, 1997; Masson, Mestre, & Stone, 1999; Reisbeck & Gegenfurtner, 1999; Verghese & McKee, 2006; Champion & Warren, 2017), almost all these studies tested speed discrimination only between motions in the same direction. One exception is the work of Matthews, Luber, Qian, and Lisanby (2001) who asked participants to compare either speed or direction in random dot patterns which differed in both dimensions. Interestingly, they found that midline occipital transcranial magnetic stimulation impaired speed but not direction discrimination, suggesting that speed judgments may depend on processes that are independent of direction.

Another exception is the experiment by Verghese and McKee (2006). The main purpose of this experiment was to examine whether speed discrimination was impaired when the two motions were integrated along a common trajectory. However in one condition the field was split between two oblique motions whose directions differed by 90°, and in another the motions were parallel on either side of a vertical or horizontal boundary. Verghese and McKee reported discrimination thresholds that were equally low in the two cases. Manning, Neil, Karaminis, and Pellicano (2015) used a similar display with adults, typical and autistic children, but did not replicate Verghese and McKee's result; for all three groups, thresholds for the split oblique motions were substantially higher than for the parallel (vertical or horizontal) motions. The interpretation of the results from both these studies is complicated by the fact that as well as difference in the relation between the directions of the motions being compared, the conditions also differed in the axis of motion, which was one of the cardinal axes in the parallel case, but oblique in the “split” case. The present study, therefore, used an experimental design in which the effects of both absolute direction (cardinal or oblique) and relative direction could be examined.

Accordingly, we tested both whether discrimination is affected when the speeds to be discriminated are in different directions, and whether speed discrimination is affected by the overall axis of motion. The “oblique effect,” poorer performance in oblique orientations compared to horizontal or vertical (Appelle, 1972), is very widespread in visual spatial discriminations, being found for acuity and contrast sensitivity (Campbell, Kulikowski, & Levinson, 1966) and many spatial judgments (Westheimer, 2003). In the area of visual motion, several reports find that direction discrimination is poorer for oblique directions (Gros, Blake, & Hiris, 1998; Dakin, Mareschal, & Bex, 2005) but that speed discrimination thresholds show no effect of direction (Matthews & Qian, 1999; Westheimer, 2003). This dissociation has implications for the way in which speed and direction information is extracted from the array of motion detectors, and the present datasets can provide extensive information on meridional variation in speed discrimination.

Most of the studies cited above have been based on data from a small number of highly practiced observers. However, there are substantial individual variations in motion discrimination performance (Halpern, Andrews, & Purves, 1999; Bosten et al., 2017), which may account for discrepancies such as those between the 2–3 observers in each of Verghese and McKee's (2006) experiments and the larger group in Manning et al. (2015). In the present study we chose to represent such variations, and minimize their effect, by testing a relatively large group of young adult participants who were psychophysically naïve, but were well familiarized with the discrimination task before data was collected for the analysis. Across four experiments, participants were asked to discriminate the speed of random-dot patches that moved either in the same direction or different directions. While our main research question focused on sensitivity to speed information, our design also afforded the opportunity to explore direction-dependent biases in speed discrimination.

## General methods

### 

#### Participants

Participants aged 18 to 40 years with no history of developmental disorders and self-reported normal or corrected-to-normal vision were recruited from the University and community. There were 12 participants in the dataset for Experiment 1 (nine males, three females, *M* = 21.75 years, *SD* = 3.55 years) and Experiment 2 (eight males, four females, *M* = 21.67 years, *SD* = 2.46), 11 participants in Experiment 3 (seven males, four females, *M* = 20.91 years, *SD* = 0.30) and 30 participants in Experiment 4 (14 males, 16 females, *M* = 27.03 years, *SD* = 5.31), with no overlap between experiments. Additional participants were excluded from the datasets due to incorrect responses on 10% or more of catch trials (Experiment 2: *n* = 2; Experiment 4: *n* = 4), or implausibly steep, step-like fitted psychometric functions (i.e., Weber fractions = 0) in one or two conditions (Experiment 3: *n* = 1; Experiment 4: *n* = 1).

#### Apparatus and stimuli

**Figure 2:**
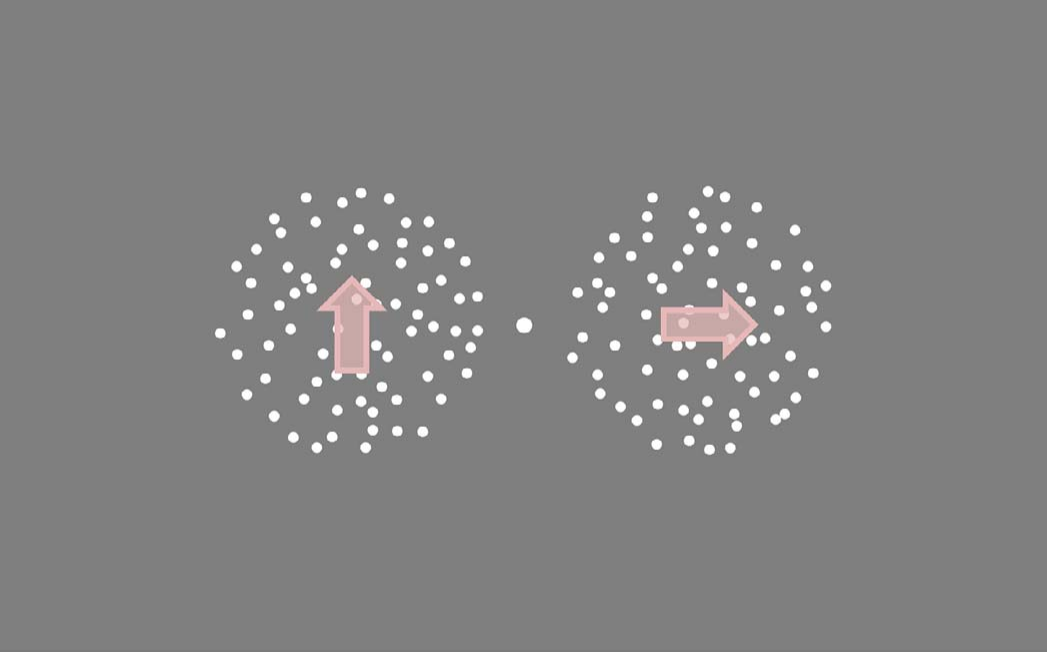
Schematic representation of stimuli. Dots moved behind circular apertures either side of a central fixation point. Arrows represent the direction of dot motion for the reference (left) and comparison (right) stimuli in the +90° direction difference condition with an upwards reference. Note that this is just one of multiple conditions presented in Experiments 1 to 4.

Stimuli were presented on a MacBook Pro laptop (2,560 × 1,600 pixels, 60 Hz) for Experiments 1 through 3 and a Dell Precision M4600 laptop (2,048 × 1,152 pixels, 60 Hz) for Experiment 4. Stimuli were presented using MATLAB (MathWorks, Natick, MA) and elements of the Psychophysics Toolbox (Brainard, 1997; Pelli, 1997; Kleiner, Brainard, & Pelli, 2007). The screen was gray with a white central fixation dot (diameter 0.30°). Stimuli were two sets of 160 white dots (diameter 0.25°) presented for 300 ms behind circular apertures (diameter 8.49°) either side of the fixation dot, separated by 2° (see Figure 2). The position of the dots updated every three screen refreshes, and each dot had a limited lifetime of 100 ms (six screen refreshes).

#### Procedure

The procedure was approved by the Central University Research Ethics Committee at the University of Oxford, in accordance with principles of the Declaration of Helsinki, and participants gave their informed consent. Participants sat at a viewing distance of 50 cm from the screen, without a chin-rest. The entire session took no longer than 1 hour.

Participants initiated each trial with the space bar and responded whether the left or right set of dots was judged to move faster using the arrow keys. On each trial, one stimulus was a reference stimulus which always moved at 6°/s, while the other stimulus was a comparison stimulus. The direction of the reference and comparison stimuli varied across conditions, with the comparison stimulus moving at an angular separation relative to the reference (see Table 1), and the location of the reference stimulus (left/right of fixation) varied randomly. The fixation point remained on the screen throughout.

**Table 1:**
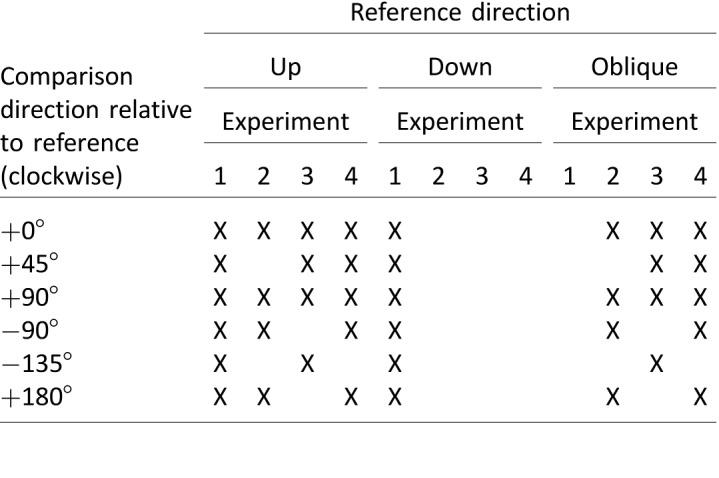
Reference and comparison directions presented in Experiments 1 to 4. Notes: Oblique reference direction refers to motion towards the top-right corner of the screen at a 45° angle.

Participants completed a criterion and a practice phase before the experimental phase. The criterion phase consisted of up to 20 trials with a comparison speed of 12°/s where participants were required to respond correctly to four consecutive trials in order to proceed to the next phase. In the criterion phase, the directions of the reference and comparison stimuli were randomly selected from the experimental conditions (see Table 1). All participants passed this criterion.

In the practice and experimental phases, the comparison speeds were determined by QUEST (Watson & Pelli, 1983). All QUEST tracks had a beta value of 3.5 (corresponding to the slope of the Weibull function assumed by QUEST; Watson & Pelli, 1983) and a lapse rate of 0.02. A random “jitter,” randomly selected from a normal distribution centred on zero with a standard deviation of 0.5°/s, was added to the values generated by QUEST.

The practice phase consisted of two randomly interleaved QUEST tracks of 20 trials for each relative direction of the comparison stimulus (one starting 3°/s above the reference speed, and one starting 3°/s below the reference speed) and an additional two easy catch trials per track with a comparison speed of 12°/s. The reference direction was randomized across trials. In total, there were 264, 176, 176, and 220 practice trials for Experiments 1 through 4, respectively. In the practice trials, the central fixation point turned red for incorrect responses. The practice phase was divided into two blocks, at which point participants were invited to take a break. A small pie chart was presented in the top left corner of the screen to indicate participants' progress through the trials.

The experimental phase consisted of four randomly interleaved QUEST tracks for each relative direction of the comparison stimulus, combining two reference directions and two starting points (3°/s above and below reference speed). The QUEST tracks each consisted of 40 trials (and two additional catch trials). Thus, there were 1,008, 672, 672, and 840 experimental trials for Experiments 1 through 4, respectively. The trials were divided into 10 blocks. Participants were not presented with feedback for incorrect responses in the experimental phase, but were provided with their average accuracy (%) at the end of each block. As in the practice phase, a pie chart was presented to indicate progress.

#### Data analysis

Cumulative Gaussian functions were fit to each individual's data (including catch trials) separately for each combination of reference direction and relative direction difference on a linearly spaced scale using the Maximum Likelihood method (Watson, 1979). The trials from QUEST tracks starting above and below the reference speed were combined (King-Smith, Grigsby, Vingrys, Benes, & Supowit, 1994). The mean of the cumulative Gaussian function was taken as the point of subjective equality (PSE), reflecting bias. The standard deviation of the cumulative Gaussian—corresponding to 84% faster judgments—was converted to a Weber fraction by dividing by the reference speed, and used as a measure of sensitivity. The data were well fit by the psychometric functions in all experiments, reflected in high R^2^ values (Experiment 1: *M* = 0.92, *SD* = 0.09; Experiment 2: *M* = 0.90, *SD* = 0.10; Experiment 3: *M* = 0.90, *SD* = 0.09; Experiment 4: *M* = 0.86, *SD* = 0.15). For all analyses, Greenhouse-Geisser corrections were applied when data violated assumptions of sphericity. The data and analysis code are available at https://osf.io/audxh/.

## Experiment 1

The primary aim of Experiment 1 was to investigate whether people's speed judgments were poorer when discriminating stimuli moving in different directions, compared to stimuli moving in the same direction. To this end, we presented participants with six direction difference conditions in which we manipulated the direction of the comparison stimulus relative to the reference (+0°, +45°, +90°, −90°, −135°, and +180°, in a clockwise direction), for both upwards and downwards reference directions (see Table 1). An additional, secondary aim was to explore possible directional biases in these speed comparisons.

## Results

**Figure 3:**
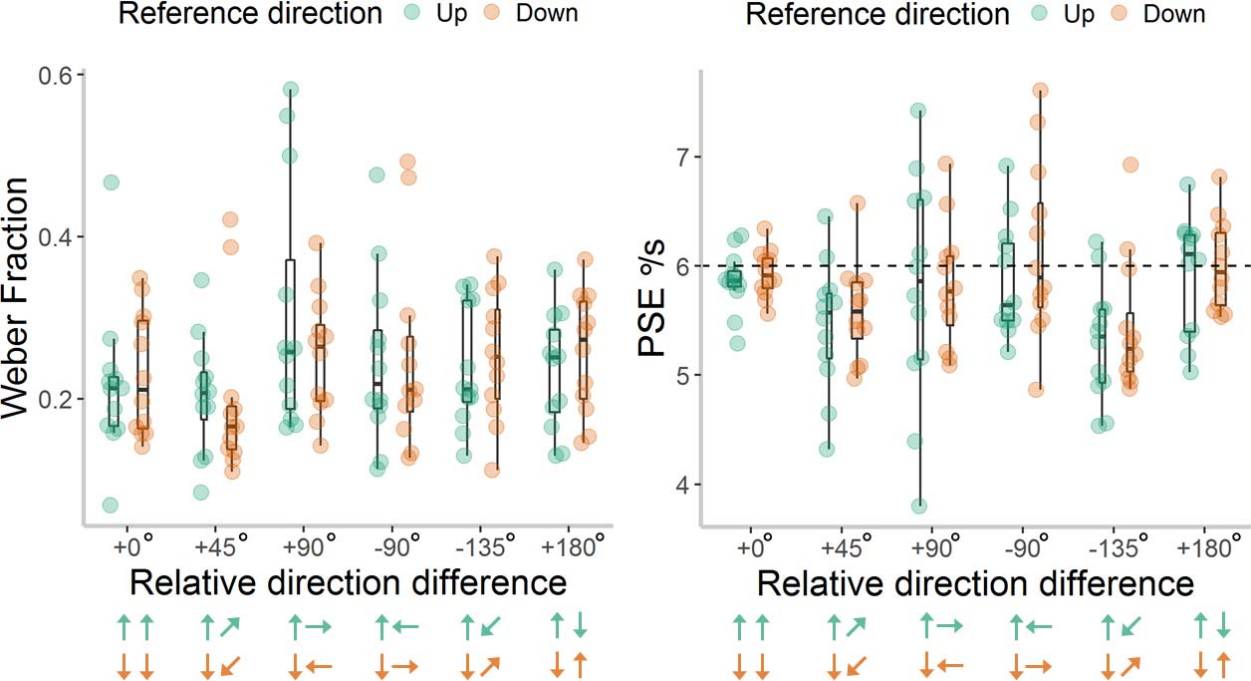
Weber Fractions and Point of Subjective Equality (PSE) values in Experiment 1. Individual Weber Fractions (left panel) and PSE values (right panel) for each reference direction and relative direction difference presented in Experiment 1. Box plots show the median, 25th and 75th percentiles of estimates, and the whiskers extend up to 1.5 times the interquartile range. The area of each box reflects the density of data points within the box. Note that lower Weber fractions reflect greater sensitivity. The dashed, horizontal line at 6°/s for the PSE values reflects veridical perception. The arrows represent the direction of the reference and comparison stimuli for each reference direction (green = up; orange = down), but note that the location of the reference stimulus was randomized between left and right in the experiment.

Weber fractions and PSE values for each participant are shown in Figure 3. To address our primary aim, we first investigated whether sensitivity varied according to the direction of stimuli, by running a 2 (reference direction) by 6 (relative direction difference) repeated measures ANOVA on Weber fractions. There was no significant effect of up versus down reference direction on Weber fractions, *F*(1, 11) < 0.01, *p* = 1.00, $\newcommand{\bialpha}{\boldsymbol{\alpha}}\newcommand{\bibeta}{\boldsymbol{\beta}}\newcommand{\bigamma}{\boldsymbol{\gamma}}\newcommand{\bidelta}{\boldsymbol{\delta}}\newcommand{\bivarepsilon}{\boldsymbol{\varepsilon}}\newcommand{\bizeta}{\boldsymbol{\zeta}}\newcommand{\bieta}{\boldsymbol{\eta}}\newcommand{\bitheta}{\boldsymbol{\theta}}\newcommand{\biiota}{\boldsymbol{\iota}}\newcommand{\bikappa}{\boldsymbol{\kappa}}\newcommand{\bilambda}{\boldsymbol{\lambda}}\newcommand{\bimu}{\boldsymbol{\mu}}\newcommand{\binu}{\boldsymbol{\nu}}\newcommand{\bixi}{\boldsymbol{\xi}}\newcommand{\biomicron}{\boldsymbol{\micron}}\newcommand{\bipi}{\boldsymbol{\pi}}\newcommand{\birho}{\boldsymbol{\rho}}\newcommand{\bisigma}{\boldsymbol{\sigma}}\newcommand{\bitau}{\boldsymbol{\tau}}\newcommand{\biupsilon}{\boldsymbol{\upsilon}}\newcommand{\biphi}{\boldsymbol{\phi}}\newcommand{\bichi}{\boldsymbol{\chi}}\newcommand{\bipsi}{\boldsymbol{\psi}}\newcommand{\biomega}{\boldsymbol{\omega}}\eta \rm{_p^2}$ < 0.01, nor a significant interaction between reference direction and direction difference, *F*(5, 55) = 1.19, *p* = 0.33, $\newcommand{\bialpha}{\boldsymbol{\alpha}}\newcommand{\bibeta}{\boldsymbol{\beta}}\newcommand{\bigamma}{\boldsymbol{\gamma}}\newcommand{\bidelta}{\boldsymbol{\delta}}\newcommand{\bivarepsilon}{\boldsymbol{\varepsilon}}\newcommand{\bizeta}{\boldsymbol{\zeta}}\newcommand{\bieta}{\boldsymbol{\eta}}\newcommand{\bitheta}{\boldsymbol{\theta}}\newcommand{\biiota}{\boldsymbol{\iota}}\newcommand{\bikappa}{\boldsymbol{\kappa}}\newcommand{\bilambda}{\boldsymbol{\lambda}}\newcommand{\bimu}{\boldsymbol{\mu}}\newcommand{\binu}{\boldsymbol{\nu}}\newcommand{\bixi}{\boldsymbol{\xi}}\newcommand{\biomicron}{\boldsymbol{\micron}}\newcommand{\bipi}{\boldsymbol{\pi}}\newcommand{\birho}{\boldsymbol{\rho}}\newcommand{\bisigma}{\boldsymbol{\sigma}}\newcommand{\bitau}{\boldsymbol{\tau}}\newcommand{\biupsilon}{\boldsymbol{\upsilon}}\newcommand{\biphi}{\boldsymbol{\phi}}\newcommand{\bichi}{\boldsymbol{\chi}}\newcommand{\bipsi}{\boldsymbol{\psi}}\newcommand{\biomega}{\boldsymbol{\omega}}\eta \rm{_p^2}$ = 0.10. There was a significant main effect of direction difference, *F*(5, 55) = 2.48, *p* = 0.04, $\newcommand{\bialpha}{\boldsymbol{\alpha}}\newcommand{\bibeta}{\boldsymbol{\beta}}\newcommand{\bigamma}{\boldsymbol{\gamma}}\newcommand{\bidelta}{\boldsymbol{\delta}}\newcommand{\bivarepsilon}{\boldsymbol{\varepsilon}}\newcommand{\bizeta}{\boldsymbol{\zeta}}\newcommand{\bieta}{\boldsymbol{\eta}}\newcommand{\bitheta}{\boldsymbol{\theta}}\newcommand{\biiota}{\boldsymbol{\iota}}\newcommand{\bikappa}{\boldsymbol{\kappa}}\newcommand{\bilambda}{\boldsymbol{\lambda}}\newcommand{\bimu}{\boldsymbol{\mu}}\newcommand{\binu}{\boldsymbol{\nu}}\newcommand{\bixi}{\boldsymbol{\xi}}\newcommand{\biomicron}{\boldsymbol{\micron}}\newcommand{\bipi}{\boldsymbol{\pi}}\newcommand{\birho}{\boldsymbol{\rho}}\newcommand{\bisigma}{\boldsymbol{\sigma}}\newcommand{\bitau}{\boldsymbol{\tau}}\newcommand{\biupsilon}{\boldsymbol{\upsilon}}\newcommand{\biphi}{\boldsymbol{\phi}}\newcommand{\bichi}{\boldsymbol{\chi}}\newcommand{\bipsi}{\boldsymbol{\psi}}\newcommand{\biomega}{\boldsymbol{\omega}}\eta \rm{_p^2}$ = 0.18. However, it was not the case that participants were more sensitive to stimuli moving in the same direction (+0°) than to stimuli moving in different directions (all *p* ≥ 0.11). Instead, repeated posthoc contrasts revealed an unpredicted significant difference in sensitivity between the +45° and +90° direction difference conditions, *F*(1, 11) = 18.70, *p* < 0.001, $\newcommand{\bialpha}{\boldsymbol{\alpha}}\newcommand{\bibeta}{\boldsymbol{\beta}}\newcommand{\bigamma}{\boldsymbol{\gamma}}\newcommand{\bidelta}{\boldsymbol{\delta}}\newcommand{\bivarepsilon}{\boldsymbol{\varepsilon}}\newcommand{\bizeta}{\boldsymbol{\zeta}}\newcommand{\bieta}{\boldsymbol{\eta}}\newcommand{\bitheta}{\boldsymbol{\theta}}\newcommand{\biiota}{\boldsymbol{\iota}}\newcommand{\bikappa}{\boldsymbol{\kappa}}\newcommand{\bilambda}{\boldsymbol{\lambda}}\newcommand{\bimu}{\boldsymbol{\mu}}\newcommand{\binu}{\boldsymbol{\nu}}\newcommand{\bixi}{\boldsymbol{\xi}}\newcommand{\biomicron}{\boldsymbol{\micron}}\newcommand{\bipi}{\boldsymbol{\pi}}\newcommand{\birho}{\boldsymbol{\rho}}\newcommand{\bisigma}{\boldsymbol{\sigma}}\newcommand{\bitau}{\boldsymbol{\tau}}\newcommand{\biupsilon}{\boldsymbol{\upsilon}}\newcommand{\biphi}{\boldsymbol{\phi}}\newcommand{\bichi}{\boldsymbol{\chi}}\newcommand{\bipsi}{\boldsymbol{\psi}}\newcommand{\biomega}{\boldsymbol{\omega}}\eta \rm{_p^2}$ = 0.63, with lower Weber fractions (i.e., higher sensitivity) in the +45° (*M* = 0.20, *SE* = 0.02) condition than in the +90° condition (*M* = 0.28, *SE* = 0.03).

To investigate possible directional biases, we conducted a 6 × 2 ANOVA on PSE values. Again, there was no significant effect of reference direction, *F*(1, 11) = 0.76, *p* = 0.40, $\newcommand{\bialpha}{\boldsymbol{\alpha}}\newcommand{\bibeta}{\boldsymbol{\beta}}\newcommand{\bigamma}{\boldsymbol{\gamma}}\newcommand{\bidelta}{\boldsymbol{\delta}}\newcommand{\bivarepsilon}{\boldsymbol{\varepsilon}}\newcommand{\bizeta}{\boldsymbol{\zeta}}\newcommand{\bieta}{\boldsymbol{\eta}}\newcommand{\bitheta}{\boldsymbol{\theta}}\newcommand{\biiota}{\boldsymbol{\iota}}\newcommand{\bikappa}{\boldsymbol{\kappa}}\newcommand{\bilambda}{\boldsymbol{\lambda}}\newcommand{\bimu}{\boldsymbol{\mu}}\newcommand{\binu}{\boldsymbol{\nu}}\newcommand{\bixi}{\boldsymbol{\xi}}\newcommand{\biomicron}{\boldsymbol{\micron}}\newcommand{\bipi}{\boldsymbol{\pi}}\newcommand{\birho}{\boldsymbol{\rho}}\newcommand{\bisigma}{\boldsymbol{\sigma}}\newcommand{\bitau}{\boldsymbol{\tau}}\newcommand{\biupsilon}{\boldsymbol{\upsilon}}\newcommand{\biphi}{\boldsymbol{\phi}}\newcommand{\bichi}{\boldsymbol{\chi}}\newcommand{\bipsi}{\boldsymbol{\psi}}\newcommand{\biomega}{\boldsymbol{\omega}}\eta \rm{_p^2}$ = 0.07, nor a significant interaction between reference direction and direction difference, *F*(5, 55) = 0.20, *p* = 0.96, $\newcommand{\bialpha}{\boldsymbol{\alpha}}\newcommand{\bibeta}{\boldsymbol{\beta}}\newcommand{\bigamma}{\boldsymbol{\gamma}}\newcommand{\bidelta}{\boldsymbol{\delta}}\newcommand{\bivarepsilon}{\boldsymbol{\varepsilon}}\newcommand{\bizeta}{\boldsymbol{\zeta}}\newcommand{\bieta}{\boldsymbol{\eta}}\newcommand{\bitheta}{\boldsymbol{\theta}}\newcommand{\biiota}{\boldsymbol{\iota}}\newcommand{\bikappa}{\boldsymbol{\kappa}}\newcommand{\bilambda}{\boldsymbol{\lambda}}\newcommand{\bimu}{\boldsymbol{\mu}}\newcommand{\binu}{\boldsymbol{\nu}}\newcommand{\bixi}{\boldsymbol{\xi}}\newcommand{\biomicron}{\boldsymbol{\micron}}\newcommand{\bipi}{\boldsymbol{\pi}}\newcommand{\birho}{\boldsymbol{\rho}}\newcommand{\bisigma}{\boldsymbol{\sigma}}\newcommand{\bitau}{\boldsymbol{\tau}}\newcommand{\biupsilon}{\boldsymbol{\upsilon}}\newcommand{\biphi}{\boldsymbol{\phi}}\newcommand{\bichi}{\boldsymbol{\chi}}\newcommand{\bipsi}{\boldsymbol{\psi}}\newcommand{\biomega}{\boldsymbol{\omega}}\eta \rm{_p^2}$ = 0.02. Interestingly, there was a significant main effect of direction difference on PSE values, *F*(5, 55) = 6.19, *p* < 0.001, $\newcommand{\bialpha}{\boldsymbol{\alpha}}\newcommand{\bibeta}{\boldsymbol{\beta}}\newcommand{\bigamma}{\boldsymbol{\gamma}}\newcommand{\bidelta}{\boldsymbol{\delta}}\newcommand{\bivarepsilon}{\boldsymbol{\varepsilon}}\newcommand{\bizeta}{\boldsymbol{\zeta}}\newcommand{\bieta}{\boldsymbol{\eta}}\newcommand{\bitheta}{\boldsymbol{\theta}}\newcommand{\biiota}{\boldsymbol{\iota}}\newcommand{\bikappa}{\boldsymbol{\kappa}}\newcommand{\bilambda}{\boldsymbol{\lambda}}\newcommand{\bimu}{\boldsymbol{\mu}}\newcommand{\binu}{\boldsymbol{\nu}}\newcommand{\bixi}{\boldsymbol{\xi}}\newcommand{\biomicron}{\boldsymbol{\micron}}\newcommand{\bipi}{\boldsymbol{\pi}}\newcommand{\birho}{\boldsymbol{\rho}}\newcommand{\bisigma}{\boldsymbol{\sigma}}\newcommand{\bitau}{\boldsymbol{\tau}}\newcommand{\biupsilon}{\boldsymbol{\upsilon}}\newcommand{\biphi}{\boldsymbol{\phi}}\newcommand{\bichi}{\boldsymbol{\chi}}\newcommand{\bipsi}{\boldsymbol{\psi}}\newcommand{\biomega}{\boldsymbol{\omega}}\eta \rm{_p^2}$ = 0.36. Planned contrasts revealed that PSE values were significantly lower when the comparison was moving at +45°: *M* = 5.52, *SE* = 0.12, *F*(1, 11) = 6.10, *p* = 0.03, $\newcommand{\bialpha}{\boldsymbol{\alpha}}\newcommand{\bibeta}{\boldsymbol{\beta}}\newcommand{\bigamma}{\boldsymbol{\gamma}}\newcommand{\bidelta}{\boldsymbol{\delta}}\newcommand{\bivarepsilon}{\boldsymbol{\varepsilon}}\newcommand{\bizeta}{\boldsymbol{\zeta}}\newcommand{\bieta}{\boldsymbol{\eta}}\newcommand{\bitheta}{\boldsymbol{\theta}}\newcommand{\biiota}{\boldsymbol{\iota}}\newcommand{\bikappa}{\boldsymbol{\kappa}}\newcommand{\bilambda}{\boldsymbol{\lambda}}\newcommand{\bimu}{\boldsymbol{\mu}}\newcommand{\binu}{\boldsymbol{\nu}}\newcommand{\bixi}{\boldsymbol{\xi}}\newcommand{\biomicron}{\boldsymbol{\micron}}\newcommand{\bipi}{\boldsymbol{\pi}}\newcommand{\birho}{\boldsymbol{\rho}}\newcommand{\bisigma}{\boldsymbol{\sigma}}\newcommand{\bitau}{\boldsymbol{\tau}}\newcommand{\biupsilon}{\boldsymbol{\upsilon}}\newcommand{\biphi}{\boldsymbol{\phi}}\newcommand{\bichi}{\boldsymbol{\chi}}\newcommand{\bipsi}{\boldsymbol{\psi}}\newcommand{\biomega}{\boldsymbol{\omega}}\eta \rm{_p^2}$ = 0.36; or −135°: *M* = 5.37, *SE* = 0.11, *F*(1, 11) = 19.18, *p* = 0.001, $\newcommand{\bialpha}{\boldsymbol{\alpha}}\newcommand{\bibeta}{\boldsymbol{\beta}}\newcommand{\bigamma}{\boldsymbol{\gamma}}\newcommand{\bidelta}{\boldsymbol{\delta}}\newcommand{\bivarepsilon}{\boldsymbol{\varepsilon}}\newcommand{\bizeta}{\boldsymbol{\zeta}}\newcommand{\bieta}{\boldsymbol{\eta}}\newcommand{\bitheta}{\boldsymbol{\theta}}\newcommand{\biiota}{\boldsymbol{\iota}}\newcommand{\bikappa}{\boldsymbol{\kappa}}\newcommand{\bilambda}{\boldsymbol{\lambda}}\newcommand{\bimu}{\boldsymbol{\mu}}\newcommand{\binu}{\boldsymbol{\nu}}\newcommand{\bixi}{\boldsymbol{\xi}}\newcommand{\biomicron}{\boldsymbol{\micron}}\newcommand{\bipi}{\boldsymbol{\pi}}\newcommand{\birho}{\boldsymbol{\rho}}\newcommand{\bisigma}{\boldsymbol{\sigma}}\newcommand{\bitau}{\boldsymbol{\tau}}\newcommand{\biupsilon}{\boldsymbol{\upsilon}}\newcommand{\biphi}{\boldsymbol{\phi}}\newcommand{\bichi}{\boldsymbol{\chi}}\newcommand{\bipsi}{\boldsymbol{\psi}}\newcommand{\biomega}{\boldsymbol{\omega}}\eta \rm{_p^2}$ = 0.64, relative to the reference, compared to when the reference moved in the same direction as the reference, in the +0° direction difference condition (*M* = 5.90, *SE* = 0.07). Therefore, comparison stimuli moving at +45° or −135° relative to the reference were perceived as moving faster than comparison stimuli moving in the same direction as the vertical reference. In contrast, there were no significant differences in PSE values between the +0° condition and the other direction difference conditions (+90°, −90°, +180°), *p* ≥ 0.56. This pattern of results was confirmed using one-sample *t* tests, which showed that the PSE values for the +45° and −135° direction difference conditions were significantly lower than veridical (6°/s), when the reference was either upwards, +45°: *t*(11) = 3.22, *p* = 0.008; −135°: *t*(11) = 4.49, *p* = 0.001, or downwards, +45°: *t*(11) = 3.24, *p* = 0.008; −135°: *t*(11) = 3.17, *p* = 0.009, whereas the PSE values in all other direction difference conditions did not differ significantly from 6°/s (*p* ≥ 0.10). Therefore, there appears to be a consistent bias for perceiving obliquely moving stimuli as faster than either upwards or downwards vertically moving stimuli.

The results of Experiment 1 did not clearly show that people are less sensitive to speed differences when comparing across stimuli in different directions. Instead, there was an unpredicted difference in sensitivity between the +45° and +90° direction difference conditions. While it is possible that sensitivity was reduced in the +90° direction difference condition compared to the +45° direction difference condition due to increased angular separation between the reference and comparison stimulus, we did not find generally reduced performance for other direction difference conditions with wide angular separation (−90°, −135°, +180°). We also found that obliquely moving stimuli were perceived as moving faster than vertically moving stimuli.

## Experiment 2

In Experiment 2, we aimed to follow up the findings from Experiment 1 of differences in speed perception for oblique and vertical directions, by testing for an oblique effect in sensitivity (i.e., reduced sensitivity to motion on the oblique compared to cardinal axes), and to provide a further test of reduced sensitivity when comparing across stimuli moving in different directions. We presented comparison stimuli that moved in the same direction as the reference (+0°), and comparison stimuli that moved +90°, −90°, and +180° relative to the reference. When the reference direction was upwards, all comparison stimuli moved in cardinal directions, and when the reference direction was oblique, all comparison stimuli moved in oblique directions. An oblique effect would therefore be reflected in reduced sensitivity to the oblique reference direction compared to the upwards reference direction. We did not expect to reveal any biases in speed perception, as we were not presenting stimuli which required comparison across cardinal and oblique directions.

## Results

**Figure 4:**
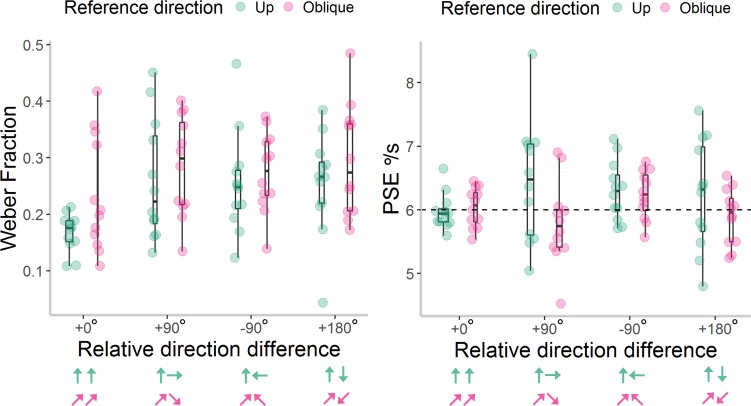
Weber Fractions and Point of Subjective Equality (PSE) values in Experiment 2. Individual Weber Fractions (left panel) and PSE values (right panel) for each reference direction and relative direction difference presented in Experiment 2. Box plots show the median, 25th and 75th percentiles of estimates, and the whiskers extend up to 1.5 times the interquartile range. The area of each box reflects the density of data points within the box. The dashed, horizontal line at 6°/s for the PSE values reflects veridical perception. The arrows represent the direction of the reference and comparison stimuli for each reference direction (green = up; pink = oblique), but note that the location of the reference stimulus was randomized between left and right in the experiment.

First, we conducted a 2 × 4 ANOVA on Weber fractions (Figure 4). There was an “oblique effect” in sensitivity, reflected in a main effect of reference direction, *F*(1, 11) = 11.12, *p* = 0.007, $\newcommand{\bialpha}{\boldsymbol{\alpha}}\newcommand{\bibeta}{\boldsymbol{\beta}}\newcommand{\bigamma}{\boldsymbol{\gamma}}\newcommand{\bidelta}{\boldsymbol{\delta}}\newcommand{\bivarepsilon}{\boldsymbol{\varepsilon}}\newcommand{\bizeta}{\boldsymbol{\zeta}}\newcommand{\bieta}{\boldsymbol{\eta}}\newcommand{\bitheta}{\boldsymbol{\theta}}\newcommand{\biiota}{\boldsymbol{\iota}}\newcommand{\bikappa}{\boldsymbol{\kappa}}\newcommand{\bilambda}{\boldsymbol{\lambda}}\newcommand{\bimu}{\boldsymbol{\mu}}\newcommand{\binu}{\boldsymbol{\nu}}\newcommand{\bixi}{\boldsymbol{\xi}}\newcommand{\biomicron}{\boldsymbol{\micron}}\newcommand{\bipi}{\boldsymbol{\pi}}\newcommand{\birho}{\boldsymbol{\rho}}\newcommand{\bisigma}{\boldsymbol{\sigma}}\newcommand{\bitau}{\boldsymbol{\tau}}\newcommand{\biupsilon}{\boldsymbol{\upsilon}}\newcommand{\biphi}{\boldsymbol{\phi}}\newcommand{\bichi}{\boldsymbol{\chi}}\newcommand{\bipsi}{\boldsymbol{\psi}}\newcommand{\biomega}{\boldsymbol{\omega}}\eta \rm{_p^2}$ = 0.50, with the upwards reference direction leading to lower Weber fractions (*M* = 0.23, *SE* = 0.02) than the oblique reference direction (*M* = 0.27, *SE* = 0.02). Additionally, there was a significant effect of direction difference on Weber fractions, *F*(3, 33) = 4.65, *p* = 0.008, $\newcommand{\bialpha}{\boldsymbol{\alpha}}\newcommand{\bibeta}{\boldsymbol{\beta}}\newcommand{\bigamma}{\boldsymbol{\gamma}}\newcommand{\bidelta}{\boldsymbol{\delta}}\newcommand{\bivarepsilon}{\boldsymbol{\varepsilon}}\newcommand{\bizeta}{\boldsymbol{\zeta}}\newcommand{\bieta}{\boldsymbol{\eta}}\newcommand{\bitheta}{\boldsymbol{\theta}}\newcommand{\biiota}{\boldsymbol{\iota}}\newcommand{\bikappa}{\boldsymbol{\kappa}}\newcommand{\bilambda}{\boldsymbol{\lambda}}\newcommand{\bimu}{\boldsymbol{\mu}}\newcommand{\binu}{\boldsymbol{\nu}}\newcommand{\bixi}{\boldsymbol{\xi}}\newcommand{\biomicron}{\boldsymbol{\micron}}\newcommand{\bipi}{\boldsymbol{\pi}}\newcommand{\birho}{\boldsymbol{\rho}}\newcommand{\bisigma}{\boldsymbol{\sigma}}\newcommand{\bitau}{\boldsymbol{\tau}}\newcommand{\biupsilon}{\boldsymbol{\upsilon}}\newcommand{\biphi}{\boldsymbol{\phi}}\newcommand{\bichi}{\boldsymbol{\chi}}\newcommand{\bipsi}{\boldsymbol{\psi}}\newcommand{\biomega}{\boldsymbol{\omega}}\eta \rm{_p^2}$ = 0.30. Planned contrasts revealed that participants had significantly lower Weber fractions when the reference and comparison stimuli moved in the same direction (+0°: *M* = 0.20, *SE* = 0.02), compared to the other three direction difference conditions (+90°: *M* = 0.27, *SE* = 0.02, *p* = 0.007; −90°: *M* = 0.26, *SE* = 0.02, *p* = 0.02; +180°: *M* = 0.27, *SE* = 0.02, *p* = 0.03). We found no significant interaction between reference direction and direction difference on Weber fractions, *F*(3, 33) = 0.63, *p* = 0.60, $\newcommand{\bialpha}{\boldsymbol{\alpha}}\newcommand{\bibeta}{\boldsymbol{\beta}}\newcommand{\bigamma}{\boldsymbol{\gamma}}\newcommand{\bidelta}{\boldsymbol{\delta}}\newcommand{\bivarepsilon}{\boldsymbol{\varepsilon}}\newcommand{\bizeta}{\boldsymbol{\zeta}}\newcommand{\bieta}{\boldsymbol{\eta}}\newcommand{\bitheta}{\boldsymbol{\theta}}\newcommand{\biiota}{\boldsymbol{\iota}}\newcommand{\bikappa}{\boldsymbol{\kappa}}\newcommand{\bilambda}{\boldsymbol{\lambda}}\newcommand{\bimu}{\boldsymbol{\mu}}\newcommand{\binu}{\boldsymbol{\nu}}\newcommand{\bixi}{\boldsymbol{\xi}}\newcommand{\biomicron}{\boldsymbol{\micron}}\newcommand{\bipi}{\boldsymbol{\pi}}\newcommand{\birho}{\boldsymbol{\rho}}\newcommand{\bisigma}{\boldsymbol{\sigma}}\newcommand{\bitau}{\boldsymbol{\tau}}\newcommand{\biupsilon}{\boldsymbol{\upsilon}}\newcommand{\biphi}{\boldsymbol{\phi}}\newcommand{\bichi}{\boldsymbol{\chi}}\newcommand{\bipsi}{\boldsymbol{\psi}}\newcommand{\biomega}{\boldsymbol{\omega}}\eta \rm{_p^2}$ = 0.05.

As expected, a 2 × 4 ANOVA on PSE values (Figure 4) revealed no biases in perception. There were no significant main effects of reference direction, *F*(1, 11) = 4.41, *p* = 0.06, $\newcommand{\bialpha}{\boldsymbol{\alpha}}\newcommand{\bibeta}{\boldsymbol{\beta}}\newcommand{\bigamma}{\boldsymbol{\gamma}}\newcommand{\bidelta}{\boldsymbol{\delta}}\newcommand{\bivarepsilon}{\boldsymbol{\varepsilon}}\newcommand{\bizeta}{\boldsymbol{\zeta}}\newcommand{\bieta}{\boldsymbol{\eta}}\newcommand{\bitheta}{\boldsymbol{\theta}}\newcommand{\biiota}{\boldsymbol{\iota}}\newcommand{\bikappa}{\boldsymbol{\kappa}}\newcommand{\bilambda}{\boldsymbol{\lambda}}\newcommand{\bimu}{\boldsymbol{\mu}}\newcommand{\binu}{\boldsymbol{\nu}}\newcommand{\bixi}{\boldsymbol{\xi}}\newcommand{\biomicron}{\boldsymbol{\micron}}\newcommand{\bipi}{\boldsymbol{\pi}}\newcommand{\birho}{\boldsymbol{\rho}}\newcommand{\bisigma}{\boldsymbol{\sigma}}\newcommand{\bitau}{\boldsymbol{\tau}}\newcommand{\biupsilon}{\boldsymbol{\upsilon}}\newcommand{\biphi}{\boldsymbol{\phi}}\newcommand{\bichi}{\boldsymbol{\chi}}\newcommand{\bipsi}{\boldsymbol{\psi}}\newcommand{\biomega}{\boldsymbol{\omega}}\eta \rm{_p^2}$ = 0.29, or direction difference, *F*(3, 33) = 1.61, *p* = 0.21, $\newcommand{\bialpha}{\boldsymbol{\alpha}}\newcommand{\bibeta}{\boldsymbol{\beta}}\newcommand{\bigamma}{\boldsymbol{\gamma}}\newcommand{\bidelta}{\boldsymbol{\delta}}\newcommand{\bivarepsilon}{\boldsymbol{\varepsilon}}\newcommand{\bizeta}{\boldsymbol{\zeta}}\newcommand{\bieta}{\boldsymbol{\eta}}\newcommand{\bitheta}{\boldsymbol{\theta}}\newcommand{\biiota}{\boldsymbol{\iota}}\newcommand{\bikappa}{\boldsymbol{\kappa}}\newcommand{\bilambda}{\boldsymbol{\lambda}}\newcommand{\bimu}{\boldsymbol{\mu}}\newcommand{\binu}{\boldsymbol{\nu}}\newcommand{\bixi}{\boldsymbol{\xi}}\newcommand{\biomicron}{\boldsymbol{\micron}}\newcommand{\bipi}{\boldsymbol{\pi}}\newcommand{\birho}{\boldsymbol{\rho}}\newcommand{\bisigma}{\boldsymbol{\sigma}}\newcommand{\bitau}{\boldsymbol{\tau}}\newcommand{\biupsilon}{\boldsymbol{\upsilon}}\newcommand{\biphi}{\boldsymbol{\phi}}\newcommand{\bichi}{\boldsymbol{\chi}}\newcommand{\bipsi}{\boldsymbol{\psi}}\newcommand{\biomega}{\boldsymbol{\omega}}\eta \rm{_p^2}$ = 0.13, and no interaction between reference direction and direction difference, *F*(2.15, 23.69) = 2.20, *p* = 0.13, $\newcommand{\bialpha}{\boldsymbol{\alpha}}\newcommand{\bibeta}{\boldsymbol{\beta}}\newcommand{\bigamma}{\boldsymbol{\gamma}}\newcommand{\bidelta}{\boldsymbol{\delta}}\newcommand{\bivarepsilon}{\boldsymbol{\varepsilon}}\newcommand{\bizeta}{\boldsymbol{\zeta}}\newcommand{\bieta}{\boldsymbol{\eta}}\newcommand{\bitheta}{\boldsymbol{\theta}}\newcommand{\biiota}{\boldsymbol{\iota}}\newcommand{\bikappa}{\boldsymbol{\kappa}}\newcommand{\bilambda}{\boldsymbol{\lambda}}\newcommand{\bimu}{\boldsymbol{\mu}}\newcommand{\binu}{\boldsymbol{\nu}}\newcommand{\bixi}{\boldsymbol{\xi}}\newcommand{\biomicron}{\boldsymbol{\micron}}\newcommand{\bipi}{\boldsymbol{\pi}}\newcommand{\birho}{\boldsymbol{\rho}}\newcommand{\bisigma}{\boldsymbol{\sigma}}\newcommand{\bitau}{\boldsymbol{\tau}}\newcommand{\biupsilon}{\boldsymbol{\upsilon}}\newcommand{\biphi}{\boldsymbol{\phi}}\newcommand{\bichi}{\boldsymbol{\chi}}\newcommand{\bipsi}{\boldsymbol{\psi}}\newcommand{\biomega}{\boldsymbol{\omega}}\eta \rm{_p^2}$ = 0.17.

The results from Experiment 2 suggest that speed discrimination sensitivity was reduced when comparing across different stimulus directions, unlike in Experiment 1. Additionally, the results suggest an oblique effect in speed discrimination (cf. Matthews & Qian, 1999; Westheimer, 2003).

## Experiment 3

In Experiment 3, we revisited the oblique bias apparent in Experiment 1. Experiment 1 showed that oblique motion (+45°, −135°) appeared to move faster than vertical stimuli. Yet, we did not test whether oblique motion also appears to move faster relative to horizontal stimuli. We addressed this outstanding question by presenting conditions with +45° and −135° direction differences, with an oblique reference as well as with an upwards reference, along with +0° and +90° direction difference conditions for comparison (see Table 1). If oblique motions are perceived to move faster than all cardinal directions (i.e., both vertical and horizontal), we would expect systematic biases in the +45° and −135° direction difference conditions for both reference directions. Specifically, for the oblique reference direction, we expected PSEs to be over 6°/s if the oblique reference was perceived to be moving faster than the horizontal comparison, whereas we expected PSEs to be under 6°/s for the upwards reference direction, as in Experiment 1. Thus, we predicted an interaction between reference direction and direction difference on PSE values. A further aim of this Experiment was to replicate our finding of an oblique effect (as found in Experiment 2) by comparing Weber fractions in the +0° and +90° direction difference conditions between the upwards and oblique reference directions and to again test whether participants show reduced sensitivity when comparing speeds across these different directions.

## Results

**Figure 5:**
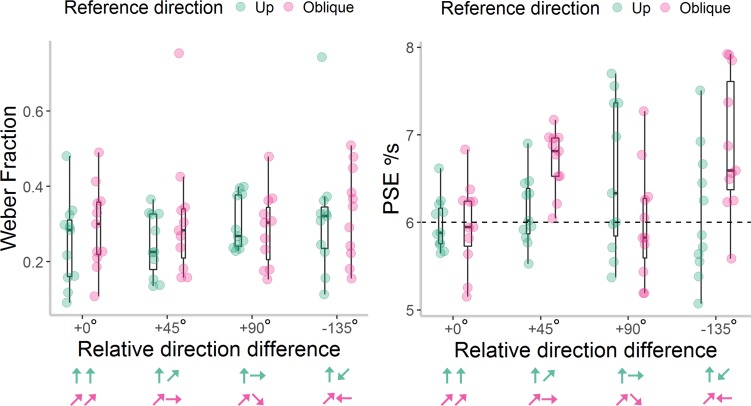
Weber Fractions and Point of Subjective Equality (PSE) values in Experiment 3. Individual Weber Fractions (left panel) and PSE values (right panel) for each reference direction and relative direction difference presented in Experiment 3. Box plots show the median, 25th and 75th percentiles of estimates, and the whiskers extend up to 1.5 times the interquartile range. The area of each box reflects the density of data points within the box. The dashed, horizontal line at 6°/s for the PSE values reflects veridical perception. The arrows represent the direction of the reference and comparison stimuli for each reference direction (green = up; pink = oblique), but note that the location of the reference stimulus was randomized between left and right in the experiment.

Here, we found no overall effect of reference direction on Weber fractions, *F*(1, 10) = 2.83, *p* = 0.12, $\newcommand{\bialpha}{\boldsymbol{\alpha}}\newcommand{\bibeta}{\boldsymbol{\beta}}\newcommand{\bigamma}{\boldsymbol{\gamma}}\newcommand{\bidelta}{\boldsymbol{\delta}}\newcommand{\bivarepsilon}{\boldsymbol{\varepsilon}}\newcommand{\bizeta}{\boldsymbol{\zeta}}\newcommand{\bieta}{\boldsymbol{\eta}}\newcommand{\bitheta}{\boldsymbol{\theta}}\newcommand{\biiota}{\boldsymbol{\iota}}\newcommand{\bikappa}{\boldsymbol{\kappa}}\newcommand{\bilambda}{\boldsymbol{\lambda}}\newcommand{\bimu}{\boldsymbol{\mu}}\newcommand{\binu}{\boldsymbol{\nu}}\newcommand{\bixi}{\boldsymbol{\xi}}\newcommand{\biomicron}{\boldsymbol{\micron}}\newcommand{\bipi}{\boldsymbol{\pi}}\newcommand{\birho}{\boldsymbol{\rho}}\newcommand{\bisigma}{\boldsymbol{\sigma}}\newcommand{\bitau}{\boldsymbol{\tau}}\newcommand{\biupsilon}{\boldsymbol{\upsilon}}\newcommand{\biphi}{\boldsymbol{\phi}}\newcommand{\bichi}{\boldsymbol{\chi}}\newcommand{\bipsi}{\boldsymbol{\psi}}\newcommand{\biomega}{\boldsymbol{\omega}}\eta \rm{_p^2}$ = 0.22, *t*(10) = 0.10, *p* = 0.92 (Figure 5). Unlike in Experiment 2, there was no significant effect of direction difference, *F*(3, 30) = 1.81, *p* = 0.17, $\newcommand{\bialpha}{\boldsymbol{\alpha}}\newcommand{\bibeta}{\boldsymbol{\beta}}\newcommand{\bigamma}{\boldsymbol{\gamma}}\newcommand{\bidelta}{\boldsymbol{\delta}}\newcommand{\bivarepsilon}{\boldsymbol{\varepsilon}}\newcommand{\bizeta}{\boldsymbol{\zeta}}\newcommand{\bieta}{\boldsymbol{\eta}}\newcommand{\bitheta}{\boldsymbol{\theta}}\newcommand{\biiota}{\boldsymbol{\iota}}\newcommand{\bikappa}{\boldsymbol{\kappa}}\newcommand{\bilambda}{\boldsymbol{\lambda}}\newcommand{\bimu}{\boldsymbol{\mu}}\newcommand{\binu}{\boldsymbol{\nu}}\newcommand{\bixi}{\boldsymbol{\xi}}\newcommand{\biomicron}{\boldsymbol{\micron}}\newcommand{\bipi}{\boldsymbol{\pi}}\newcommand{\birho}{\boldsymbol{\rho}}\newcommand{\bisigma}{\boldsymbol{\sigma}}\newcommand{\bitau}{\boldsymbol{\tau}}\newcommand{\biupsilon}{\boldsymbol{\upsilon}}\newcommand{\biphi}{\boldsymbol{\phi}}\newcommand{\bichi}{\boldsymbol{\chi}}\newcommand{\bipsi}{\boldsymbol{\psi}}\newcommand{\biomega}{\boldsymbol{\omega}}\eta \rm{_p^2}$ = 0.15, nor an interaction between reference direction and direction difference, *F*(3, 30) = 1.08, *p* = 0.37, $\newcommand{\bialpha}{\boldsymbol{\alpha}}\newcommand{\bibeta}{\boldsymbol{\beta}}\newcommand{\bigamma}{\boldsymbol{\gamma}}\newcommand{\bidelta}{\boldsymbol{\delta}}\newcommand{\bivarepsilon}{\boldsymbol{\varepsilon}}\newcommand{\bizeta}{\boldsymbol{\zeta}}\newcommand{\bieta}{\boldsymbol{\eta}}\newcommand{\bitheta}{\boldsymbol{\theta}}\newcommand{\biiota}{\boldsymbol{\iota}}\newcommand{\bikappa}{\boldsymbol{\kappa}}\newcommand{\bilambda}{\boldsymbol{\lambda}}\newcommand{\bimu}{\boldsymbol{\mu}}\newcommand{\binu}{\boldsymbol{\nu}}\newcommand{\bixi}{\boldsymbol{\xi}}\newcommand{\biomicron}{\boldsymbol{\micron}}\newcommand{\bipi}{\boldsymbol{\pi}}\newcommand{\birho}{\boldsymbol{\rho}}\newcommand{\bisigma}{\boldsymbol{\sigma}}\newcommand{\bitau}{\boldsymbol{\tau}}\newcommand{\biupsilon}{\boldsymbol{\upsilon}}\newcommand{\biphi}{\boldsymbol{\phi}}\newcommand{\bichi}{\boldsymbol{\chi}}\newcommand{\bipsi}{\boldsymbol{\psi}}\newcommand{\biomega}{\boldsymbol{\omega}}\eta \rm{_p^2}$ = 0.10. To test for an oblique effect, we compared the +0° and +90° direction difference conditions between the two reference directions in a reduced, 2 × 2 ANOVA. There was no evidence for an oblique effect, with no significant effect of reference direction, *F*(1, 10) = 0.53, *p* = 0.49, $\newcommand{\bialpha}{\boldsymbol{\alpha}}\newcommand{\bibeta}{\boldsymbol{\beta}}\newcommand{\bigamma}{\boldsymbol{\gamma}}\newcommand{\bidelta}{\boldsymbol{\delta}}\newcommand{\bivarepsilon}{\boldsymbol{\varepsilon}}\newcommand{\bizeta}{\boldsymbol{\zeta}}\newcommand{\bieta}{\boldsymbol{\eta}}\newcommand{\bitheta}{\boldsymbol{\theta}}\newcommand{\biiota}{\boldsymbol{\iota}}\newcommand{\bikappa}{\boldsymbol{\kappa}}\newcommand{\bilambda}{\boldsymbol{\lambda}}\newcommand{\bimu}{\boldsymbol{\mu}}\newcommand{\binu}{\boldsymbol{\nu}}\newcommand{\bixi}{\boldsymbol{\xi}}\newcommand{\biomicron}{\boldsymbol{\micron}}\newcommand{\bipi}{\boldsymbol{\pi}}\newcommand{\birho}{\boldsymbol{\rho}}\newcommand{\bisigma}{\boldsymbol{\sigma}}\newcommand{\bitau}{\boldsymbol{\tau}}\newcommand{\biupsilon}{\boldsymbol{\upsilon}}\newcommand{\biphi}{\boldsymbol{\phi}}\newcommand{\bichi}{\boldsymbol{\chi}}\newcommand{\bipsi}{\boldsymbol{\psi}}\newcommand{\biomega}{\boldsymbol{\omega}}\eta \rm{_p^2}$ = 0.05, nor a significant interaction between reference direction and direction difference, *F*(1, 10) = 1.06, *p* = 0.33, $\newcommand{\bialpha}{\boldsymbol{\alpha}}\newcommand{\bibeta}{\boldsymbol{\beta}}\newcommand{\bigamma}{\boldsymbol{\gamma}}\newcommand{\bidelta}{\boldsymbol{\delta}}\newcommand{\bivarepsilon}{\boldsymbol{\varepsilon}}\newcommand{\bizeta}{\boldsymbol{\zeta}}\newcommand{\bieta}{\boldsymbol{\eta}}\newcommand{\bitheta}{\boldsymbol{\theta}}\newcommand{\biiota}{\boldsymbol{\iota}}\newcommand{\bikappa}{\boldsymbol{\kappa}}\newcommand{\bilambda}{\boldsymbol{\lambda}}\newcommand{\bimu}{\boldsymbol{\mu}}\newcommand{\binu}{\boldsymbol{\nu}}\newcommand{\bixi}{\boldsymbol{\xi}}\newcommand{\biomicron}{\boldsymbol{\micron}}\newcommand{\bipi}{\boldsymbol{\pi}}\newcommand{\birho}{\boldsymbol{\rho}}\newcommand{\bisigma}{\boldsymbol{\sigma}}\newcommand{\bitau}{\boldsymbol{\tau}}\newcommand{\biupsilon}{\boldsymbol{\upsilon}}\newcommand{\biphi}{\boldsymbol{\phi}}\newcommand{\bichi}{\boldsymbol{\chi}}\newcommand{\bipsi}{\boldsymbol{\psi}}\newcommand{\biomega}{\boldsymbol{\omega}}\eta \rm{_p^2}$ = 0.10. Additionally, there was no effect of direction difference in this reduced ANOVA, *F*(1, 10) = 0.86, *p* = 0.38, $\newcommand{\bialpha}{\boldsymbol{\alpha}}\newcommand{\bibeta}{\boldsymbol{\beta}}\newcommand{\bigamma}{\boldsymbol{\gamma}}\newcommand{\bidelta}{\boldsymbol{\delta}}\newcommand{\bivarepsilon}{\boldsymbol{\varepsilon}}\newcommand{\bizeta}{\boldsymbol{\zeta}}\newcommand{\bieta}{\boldsymbol{\eta}}\newcommand{\bitheta}{\boldsymbol{\theta}}\newcommand{\biiota}{\boldsymbol{\iota}}\newcommand{\bikappa}{\boldsymbol{\kappa}}\newcommand{\bilambda}{\boldsymbol{\lambda}}\newcommand{\bimu}{\boldsymbol{\mu}}\newcommand{\binu}{\boldsymbol{\nu}}\newcommand{\bixi}{\boldsymbol{\xi}}\newcommand{\biomicron}{\boldsymbol{\micron}}\newcommand{\bipi}{\boldsymbol{\pi}}\newcommand{\birho}{\boldsymbol{\rho}}\newcommand{\bisigma}{\boldsymbol{\sigma}}\newcommand{\bitau}{\boldsymbol{\tau}}\newcommand{\biupsilon}{\boldsymbol{\upsilon}}\newcommand{\biphi}{\boldsymbol{\phi}}\newcommand{\bichi}{\boldsymbol{\chi}}\newcommand{\bipsi}{\boldsymbol{\psi}}\newcommand{\biomega}{\boldsymbol{\omega}}\eta \rm{_p^2}$ = 0.08.

As expected, there was no significant main effect of reference direction on PSE values, *F*(1, 10) = 2.03, *p* = 0.18, $\newcommand{\bialpha}{\boldsymbol{\alpha}}\newcommand{\bibeta}{\boldsymbol{\beta}}\newcommand{\bigamma}{\boldsymbol{\gamma}}\newcommand{\bidelta}{\boldsymbol{\delta}}\newcommand{\bivarepsilon}{\boldsymbol{\varepsilon}}\newcommand{\bizeta}{\boldsymbol{\zeta}}\newcommand{\bieta}{\boldsymbol{\eta}}\newcommand{\bitheta}{\boldsymbol{\theta}}\newcommand{\biiota}{\boldsymbol{\iota}}\newcommand{\bikappa}{\boldsymbol{\kappa}}\newcommand{\bilambda}{\boldsymbol{\lambda}}\newcommand{\bimu}{\boldsymbol{\mu}}\newcommand{\binu}{\boldsymbol{\nu}}\newcommand{\bixi}{\boldsymbol{\xi}}\newcommand{\biomicron}{\boldsymbol{\micron}}\newcommand{\bipi}{\boldsymbol{\pi}}\newcommand{\birho}{\boldsymbol{\rho}}\newcommand{\bisigma}{\boldsymbol{\sigma}}\newcommand{\bitau}{\boldsymbol{\tau}}\newcommand{\biupsilon}{\boldsymbol{\upsilon}}\newcommand{\biphi}{\boldsymbol{\phi}}\newcommand{\bichi}{\boldsymbol{\chi}}\newcommand{\bipsi}{\boldsymbol{\psi}}\newcommand{\biomega}{\boldsymbol{\omega}}\eta \rm{_p^2}$ = 0.17 (Figure 5). However, there was a significant main effect of direction difference, *F*(3, 30) = 3.93, *p* = 0.02, $\newcommand{\bialpha}{\boldsymbol{\alpha}}\newcommand{\bibeta}{\boldsymbol{\beta}}\newcommand{\bigamma}{\boldsymbol{\gamma}}\newcommand{\bidelta}{\boldsymbol{\delta}}\newcommand{\bivarepsilon}{\boldsymbol{\varepsilon}}\newcommand{\bizeta}{\boldsymbol{\zeta}}\newcommand{\bieta}{\boldsymbol{\eta}}\newcommand{\bitheta}{\boldsymbol{\theta}}\newcommand{\biiota}{\boldsymbol{\iota}}\newcommand{\bikappa}{\boldsymbol{\kappa}}\newcommand{\bilambda}{\boldsymbol{\lambda}}\newcommand{\bimu}{\boldsymbol{\mu}}\newcommand{\binu}{\boldsymbol{\nu}}\newcommand{\bixi}{\boldsymbol{\xi}}\newcommand{\biomicron}{\boldsymbol{\micron}}\newcommand{\bipi}{\boldsymbol{\pi}}\newcommand{\birho}{\boldsymbol{\rho}}\newcommand{\bisigma}{\boldsymbol{\sigma}}\newcommand{\bitau}{\boldsymbol{\tau}}\newcommand{\biupsilon}{\boldsymbol{\upsilon}}\newcommand{\biphi}{\boldsymbol{\phi}}\newcommand{\bichi}{\boldsymbol{\chi}}\newcommand{\bipsi}{\boldsymbol{\psi}}\newcommand{\biomega}{\boldsymbol{\omega}}\eta \rm{_p^2}$ = 0.28, and the expected significant interaction between reference direction and direction difference, *F*(3, 30) = 6.83, *p* = 0.001, $\newcommand{\bialpha}{\boldsymbol{\alpha}}\newcommand{\bibeta}{\boldsymbol{\beta}}\newcommand{\bigamma}{\boldsymbol{\gamma}}\newcommand{\bidelta}{\boldsymbol{\delta}}\newcommand{\bivarepsilon}{\boldsymbol{\varepsilon}}\newcommand{\bizeta}{\boldsymbol{\zeta}}\newcommand{\bieta}{\boldsymbol{\eta}}\newcommand{\bitheta}{\boldsymbol{\theta}}\newcommand{\biiota}{\boldsymbol{\iota}}\newcommand{\bikappa}{\boldsymbol{\kappa}}\newcommand{\bilambda}{\boldsymbol{\lambda}}\newcommand{\bimu}{\boldsymbol{\mu}}\newcommand{\binu}{\boldsymbol{\nu}}\newcommand{\bixi}{\boldsymbol{\xi}}\newcommand{\biomicron}{\boldsymbol{\micron}}\newcommand{\bipi}{\boldsymbol{\pi}}\newcommand{\birho}{\boldsymbol{\rho}}\newcommand{\bisigma}{\boldsymbol{\sigma}}\newcommand{\bitau}{\boldsymbol{\tau}}\newcommand{\biupsilon}{\boldsymbol{\upsilon}}\newcommand{\biphi}{\boldsymbol{\phi}}\newcommand{\bichi}{\boldsymbol{\chi}}\newcommand{\bipsi}{\boldsymbol{\psi}}\newcommand{\biomega}{\boldsymbol{\omega}}\eta \rm{_p^2}$ = 0.41. Posthoc one-way ANOVAs showed that direction difference was only significant for the oblique reference direction, *F*(3, 30) = 7.90, *p* < 0.001, $\newcommand{\bialpha}{\boldsymbol{\alpha}}\newcommand{\bibeta}{\boldsymbol{\beta}}\newcommand{\bigamma}{\boldsymbol{\gamma}}\newcommand{\bidelta}{\boldsymbol{\delta}}\newcommand{\bivarepsilon}{\boldsymbol{\varepsilon}}\newcommand{\bizeta}{\boldsymbol{\zeta}}\newcommand{\bieta}{\boldsymbol{\eta}}\newcommand{\bitheta}{\boldsymbol{\theta}}\newcommand{\biiota}{\boldsymbol{\iota}}\newcommand{\bikappa}{\boldsymbol{\kappa}}\newcommand{\bilambda}{\boldsymbol{\lambda}}\newcommand{\bimu}{\boldsymbol{\mu}}\newcommand{\binu}{\boldsymbol{\nu}}\newcommand{\bixi}{\boldsymbol{\xi}}\newcommand{\biomicron}{\boldsymbol{\micron}}\newcommand{\bipi}{\boldsymbol{\pi}}\newcommand{\birho}{\boldsymbol{\rho}}\newcommand{\bisigma}{\boldsymbol{\sigma}}\newcommand{\bitau}{\boldsymbol{\tau}}\newcommand{\biupsilon}{\boldsymbol{\upsilon}}\newcommand{\biphi}{\boldsymbol{\phi}}\newcommand{\bichi}{\boldsymbol{\chi}}\newcommand{\bipsi}{\boldsymbol{\psi}}\newcommand{\biomega}{\boldsymbol{\omega}}\eta \rm{_p^2}$ = 0.44, but not the upwards reference direction, *F*(3, 30) = 2.41, *p* = 0.09, $\newcommand{\bialpha}{\boldsymbol{\alpha}}\newcommand{\bibeta}{\boldsymbol{\beta}}\newcommand{\bigamma}{\boldsymbol{\gamma}}\newcommand{\bidelta}{\boldsymbol{\delta}}\newcommand{\bivarepsilon}{\boldsymbol{\varepsilon}}\newcommand{\bizeta}{\boldsymbol{\zeta}}\newcommand{\bieta}{\boldsymbol{\eta}}\newcommand{\bitheta}{\boldsymbol{\theta}}\newcommand{\biiota}{\boldsymbol{\iota}}\newcommand{\bikappa}{\boldsymbol{\kappa}}\newcommand{\bilambda}{\boldsymbol{\lambda}}\newcommand{\bimu}{\boldsymbol{\mu}}\newcommand{\binu}{\boldsymbol{\nu}}\newcommand{\bixi}{\boldsymbol{\xi}}\newcommand{\biomicron}{\boldsymbol{\micron}}\newcommand{\bipi}{\boldsymbol{\pi}}\newcommand{\birho}{\boldsymbol{\rho}}\newcommand{\bisigma}{\boldsymbol{\sigma}}\newcommand{\bitau}{\boldsymbol{\tau}}\newcommand{\biupsilon}{\boldsymbol{\upsilon}}\newcommand{\biphi}{\boldsymbol{\phi}}\newcommand{\bichi}{\boldsymbol{\chi}}\newcommand{\bipsi}{\boldsymbol{\psi}}\newcommand{\biomega}{\boldsymbol{\omega}}\eta \rm{_p^2}$ = 0.19. In the oblique condition, the +45° direction difference condition, *M* = 6.71, *SE* = 0.10, *F*(1, 10) = 19.83, *p* = 0.001, $\newcommand{\bialpha}{\boldsymbol{\alpha}}\newcommand{\bibeta}{\boldsymbol{\beta}}\newcommand{\bigamma}{\boldsymbol{\gamma}}\newcommand{\bidelta}{\boldsymbol{\delta}}\newcommand{\bivarepsilon}{\boldsymbol{\varepsilon}}\newcommand{\bizeta}{\boldsymbol{\zeta}}\newcommand{\bieta}{\boldsymbol{\eta}}\newcommand{\bitheta}{\boldsymbol{\theta}}\newcommand{\biiota}{\boldsymbol{\iota}}\newcommand{\bikappa}{\boldsymbol{\kappa}}\newcommand{\bilambda}{\boldsymbol{\lambda}}\newcommand{\bimu}{\boldsymbol{\mu}}\newcommand{\binu}{\boldsymbol{\nu}}\newcommand{\bixi}{\boldsymbol{\xi}}\newcommand{\biomicron}{\boldsymbol{\micron}}\newcommand{\bipi}{\boldsymbol{\pi}}\newcommand{\birho}{\boldsymbol{\rho}}\newcommand{\bisigma}{\boldsymbol{\sigma}}\newcommand{\bitau}{\boldsymbol{\tau}}\newcommand{\biupsilon}{\boldsymbol{\upsilon}}\newcommand{\biphi}{\boldsymbol{\phi}}\newcommand{\bichi}{\boldsymbol{\chi}}\newcommand{\bipsi}{\boldsymbol{\psi}}\newcommand{\biomega}{\boldsymbol{\omega}}\eta \rm{_p^2}$ = 0.67, and −135° direction difference condition, *M* = 6.88, *SE* = 0.24, *F*(1, 10) = 10.72, *p* = 0.008, $\newcommand{\bialpha}{\boldsymbol{\alpha}}\newcommand{\bibeta}{\boldsymbol{\beta}}\newcommand{\bigamma}{\boldsymbol{\gamma}}\newcommand{\bidelta}{\boldsymbol{\delta}}\newcommand{\bivarepsilon}{\boldsymbol{\varepsilon}}\newcommand{\bizeta}{\boldsymbol{\zeta}}\newcommand{\bieta}{\boldsymbol{\eta}}\newcommand{\bitheta}{\boldsymbol{\theta}}\newcommand{\biiota}{\boldsymbol{\iota}}\newcommand{\bikappa}{\boldsymbol{\kappa}}\newcommand{\bilambda}{\boldsymbol{\lambda}}\newcommand{\bimu}{\boldsymbol{\mu}}\newcommand{\binu}{\boldsymbol{\nu}}\newcommand{\bixi}{\boldsymbol{\xi}}\newcommand{\biomicron}{\boldsymbol{\micron}}\newcommand{\bipi}{\boldsymbol{\pi}}\newcommand{\birho}{\boldsymbol{\rho}}\newcommand{\bisigma}{\boldsymbol{\sigma}}\newcommand{\bitau}{\boldsymbol{\tau}}\newcommand{\biupsilon}{\boldsymbol{\upsilon}}\newcommand{\biphi}{\boldsymbol{\phi}}\newcommand{\bichi}{\boldsymbol{\chi}}\newcommand{\bipsi}{\boldsymbol{\psi}}\newcommand{\biomega}{\boldsymbol{\omega}}\eta \rm{_p^2}$ = 0.52, differed significantly from the +0° condition (*M* = 5.97, *SE* = 0.15), whereas the +90° direction difference condition did not, *M* = 5.99, *SE* = 0.19, *F*(1, 10) = 0.01, *p* = 0.95, $\newcommand{\bialpha}{\boldsymbol{\alpha}}\newcommand{\bibeta}{\boldsymbol{\beta}}\newcommand{\bigamma}{\boldsymbol{\gamma}}\newcommand{\bidelta}{\boldsymbol{\delta}}\newcommand{\bivarepsilon}{\boldsymbol{\varepsilon}}\newcommand{\bizeta}{\boldsymbol{\zeta}}\newcommand{\bieta}{\boldsymbol{\eta}}\newcommand{\bitheta}{\boldsymbol{\theta}}\newcommand{\biiota}{\boldsymbol{\iota}}\newcommand{\bikappa}{\boldsymbol{\kappa}}\newcommand{\bilambda}{\boldsymbol{\lambda}}\newcommand{\bimu}{\boldsymbol{\mu}}\newcommand{\binu}{\boldsymbol{\nu}}\newcommand{\bixi}{\boldsymbol{\xi}}\newcommand{\biomicron}{\boldsymbol{\micron}}\newcommand{\bipi}{\boldsymbol{\pi}}\newcommand{\birho}{\boldsymbol{\rho}}\newcommand{\bisigma}{\boldsymbol{\sigma}}\newcommand{\bitau}{\boldsymbol{\tau}}\newcommand{\biupsilon}{\boldsymbol{\upsilon}}\newcommand{\biphi}{\boldsymbol{\phi}}\newcommand{\bichi}{\boldsymbol{\chi}}\newcommand{\bipsi}{\boldsymbol{\psi}}\newcommand{\biomega}{\boldsymbol{\omega}}\eta \rm{_p^2}$ < 0.01. As expected, the PSE values in the +45° and −135° direction difference conditions for the oblique reference direction were significantly above 6°/s in one-sample *t* tests: +45: *t*(10) = 6.81, *p* < 0.001; −135°: *t*(10) = 3.71, *p* = 0.004. The other conditions for both reference directions did not differ significantly from 6°/s (*p* ≥ 0.07).

Therefore, in this experiment, participants appeared to perceive an oblique stimulus as moving faster than the horizontal, but not faster than the vertical, upwards stimulus, and were not demonstrably more sensitive to speed differences across stimuli moving in the same, compared to different directions. These results contrast the findings from the previous experiments, including the finding that oblique stimuli were perceived to be moving faster than vertical stimuli in Experiment 1 and the finding of reduced sensitivity to stimuli moving in different directions in Experiment 2 (see Figure 7 for comparison). It should also be noted that there was no clear difference in PSE values between vertical and horizontal moving comparison stimuli in Experiment 1. These discrepancies could potentially arise due to the exact set of conditions presented. For example, vertical motions were more heavily represented in Experiment 1 as both reference directions were vertical, which may have led to an adaptation effect. However, it is not immediately clear how this would affect the pattern of biases found. It is also important to consider the role of extensive individual variability, which is apparent in Figures 3 through 5. It is possible that our samples of *n* ≤ 12 were underpowered to reliably detect effects (despite being larger than many comparable studies of similar phenomena). Therefore, in Experiment 4, we aimed to replicate the main findings from Experiments 1 to 3 in a larger sample of participants.

## Experiment 4

Here, we presented five relative direction differences (+0°, +45°, +90°, −90°, +180°) and two reference directions (upwards, oblique) to test whether the effects found across Experiments 1 through 3 could be replicated in a larger sample (*n* = 30). Specifically, we aimed to (a) assess whether sensitivity is reduced when comparing speeds across different directions, compared to when comparing speeds for the same direction, (b) test for the presence of an oblique effect in Weber fractions for speed, and (c) test for biases in speed perception related to obliquely moving stimuli.

## Results

**Figure 6:**
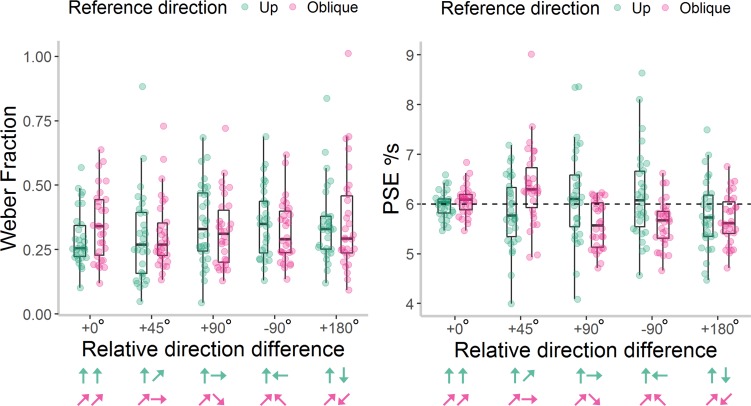
Weber Fractions and Point of Subjective Equality (PSE) values in Experiment 4. Individual Weber Fractions (left panel) and PSE values (right panel) for each reference direction and relative direction difference presented in Experiment 4. Box plots show the median, 25th and 75th percentiles of estimates, and the whiskers extend up to 1.5 times the interquartile range. The area of each box reflects the density of data points within the box. The dashed, horizontal line at 6°/s for the PSE values reflects veridical perception. The arrows represent the direction of the reference and comparison stimuli for each reference direction (green = up; pink = oblique), but note that the location of the reference stimulus was randomized between left and right in the experiment.

**Figure 7:**
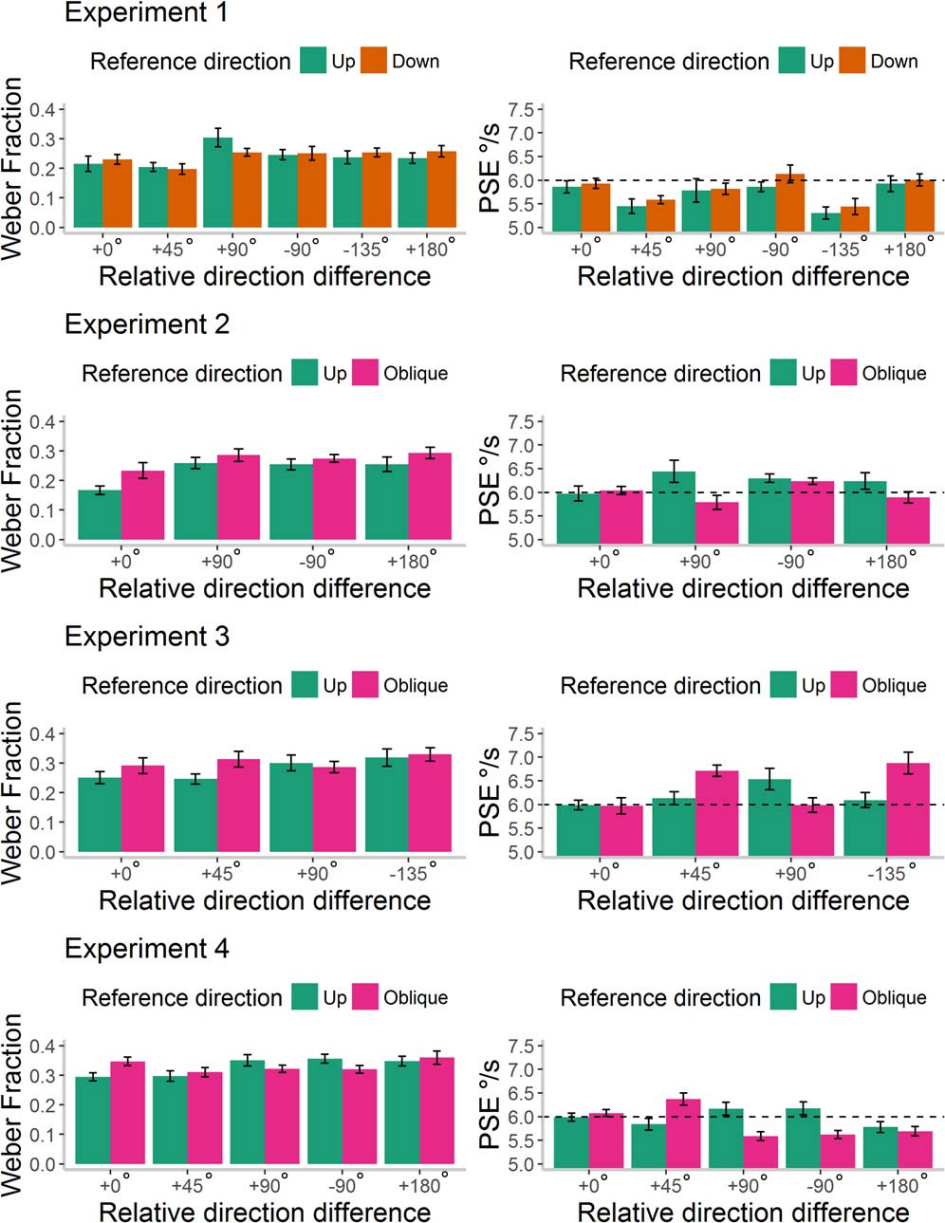
Summary of mean Weber fractions and PSE values across Experiments 1 through 4. Error bars represent ±1 SEM. Horizontal line at 6°/s for PSE values reflects veridical perception.

First, we aimed to investigate whether there was reduced sensitivity for speed discriminations made across different directions, as suggested in Experiment 2. We therefore conducted a 5 × 2 ANOVA on Weber Fractions (Figure 6). There was no overall effect of reference direction, *F*(1, 29) = 0.07, *p* = 0.80, $\newcommand{\bialpha}{\boldsymbol{\alpha}}\newcommand{\bibeta}{\boldsymbol{\beta}}\newcommand{\bigamma}{\boldsymbol{\gamma}}\newcommand{\bidelta}{\boldsymbol{\delta}}\newcommand{\bivarepsilon}{\boldsymbol{\varepsilon}}\newcommand{\bizeta}{\boldsymbol{\zeta}}\newcommand{\bieta}{\boldsymbol{\eta}}\newcommand{\bitheta}{\boldsymbol{\theta}}\newcommand{\biiota}{\boldsymbol{\iota}}\newcommand{\bikappa}{\boldsymbol{\kappa}}\newcommand{\bilambda}{\boldsymbol{\lambda}}\newcommand{\bimu}{\boldsymbol{\mu}}\newcommand{\binu}{\boldsymbol{\nu}}\newcommand{\bixi}{\boldsymbol{\xi}}\newcommand{\biomicron}{\boldsymbol{\micron}}\newcommand{\bipi}{\boldsymbol{\pi}}\newcommand{\birho}{\boldsymbol{\rho}}\newcommand{\bisigma}{\boldsymbol{\sigma}}\newcommand{\bitau}{\boldsymbol{\tau}}\newcommand{\biupsilon}{\boldsymbol{\upsilon}}\newcommand{\biphi}{\boldsymbol{\phi}}\newcommand{\bichi}{\boldsymbol{\chi}}\newcommand{\bipsi}{\boldsymbol{\psi}}\newcommand{\biomega}{\boldsymbol{\omega}}\eta \rm{_p^2}$ < 0.01, nor a significant effect of direction difference, *F*(4, 116) = 2.04, *p* = 0.10, $\newcommand{\bialpha}{\boldsymbol{\alpha}}\newcommand{\bibeta}{\boldsymbol{\beta}}\newcommand{\bigamma}{\boldsymbol{\gamma}}\newcommand{\bidelta}{\boldsymbol{\delta}}\newcommand{\bivarepsilon}{\boldsymbol{\varepsilon}}\newcommand{\bizeta}{\boldsymbol{\zeta}}\newcommand{\bieta}{\boldsymbol{\eta}}\newcommand{\bitheta}{\boldsymbol{\theta}}\newcommand{\biiota}{\boldsymbol{\iota}}\newcommand{\bikappa}{\boldsymbol{\kappa}}\newcommand{\bilambda}{\boldsymbol{\lambda}}\newcommand{\bimu}{\boldsymbol{\mu}}\newcommand{\binu}{\boldsymbol{\nu}}\newcommand{\bixi}{\boldsymbol{\xi}}\newcommand{\biomicron}{\boldsymbol{\micron}}\newcommand{\bipi}{\boldsymbol{\pi}}\newcommand{\birho}{\boldsymbol{\rho}}\newcommand{\bisigma}{\boldsymbol{\sigma}}\newcommand{\bitau}{\boldsymbol{\tau}}\newcommand{\biupsilon}{\boldsymbol{\upsilon}}\newcommand{\biphi}{\boldsymbol{\phi}}\newcommand{\bichi}{\boldsymbol{\chi}}\newcommand{\bipsi}{\boldsymbol{\psi}}\newcommand{\biomega}{\boldsymbol{\omega}}\eta \rm{_p^2}$ = 0.07; but importantly, there was a significant interaction between these two factors, *F*(3.02, 87.45) = 3.48, *p* = 0.02, $\newcommand{\bialpha}{\boldsymbol{\alpha}}\newcommand{\bibeta}{\boldsymbol{\beta}}\newcommand{\bigamma}{\boldsymbol{\gamma}}\newcommand{\bidelta}{\boldsymbol{\delta}}\newcommand{\bivarepsilon}{\boldsymbol{\varepsilon}}\newcommand{\bizeta}{\boldsymbol{\zeta}}\newcommand{\bieta}{\boldsymbol{\eta}}\newcommand{\bitheta}{\boldsymbol{\theta}}\newcommand{\biiota}{\boldsymbol{\iota}}\newcommand{\bikappa}{\boldsymbol{\kappa}}\newcommand{\bilambda}{\boldsymbol{\lambda}}\newcommand{\bimu}{\boldsymbol{\mu}}\newcommand{\binu}{\boldsymbol{\nu}}\newcommand{\bixi}{\boldsymbol{\xi}}\newcommand{\biomicron}{\boldsymbol{\micron}}\newcommand{\bipi}{\boldsymbol{\pi}}\newcommand{\birho}{\boldsymbol{\rho}}\newcommand{\bisigma}{\boldsymbol{\sigma}}\newcommand{\bitau}{\boldsymbol{\tau}}\newcommand{\biupsilon}{\boldsymbol{\upsilon}}\newcommand{\biphi}{\boldsymbol{\phi}}\newcommand{\bichi}{\boldsymbol{\chi}}\newcommand{\bipsi}{\boldsymbol{\psi}}\newcommand{\biomega}{\boldsymbol{\omega}}\eta \rm{_p^2}$ = 0.11. Posthoc one-way ANOVAs showed a significant effect of direction difference when the reference moved upwards, *F*(3.27, 94.75) = 3.36, *p* = 0.02, $\newcommand{\bialpha}{\boldsymbol{\alpha}}\newcommand{\bibeta}{\boldsymbol{\beta}}\newcommand{\bigamma}{\boldsymbol{\gamma}}\newcommand{\bidelta}{\boldsymbol{\delta}}\newcommand{\bivarepsilon}{\boldsymbol{\varepsilon}}\newcommand{\bizeta}{\boldsymbol{\zeta}}\newcommand{\bieta}{\boldsymbol{\eta}}\newcommand{\bitheta}{\boldsymbol{\theta}}\newcommand{\biiota}{\boldsymbol{\iota}}\newcommand{\bikappa}{\boldsymbol{\kappa}}\newcommand{\bilambda}{\boldsymbol{\lambda}}\newcommand{\bimu}{\boldsymbol{\mu}}\newcommand{\binu}{\boldsymbol{\nu}}\newcommand{\bixi}{\boldsymbol{\xi}}\newcommand{\biomicron}{\boldsymbol{\micron}}\newcommand{\bipi}{\boldsymbol{\pi}}\newcommand{\birho}{\boldsymbol{\rho}}\newcommand{\bisigma}{\boldsymbol{\sigma}}\newcommand{\bitau}{\boldsymbol{\tau}}\newcommand{\biupsilon}{\boldsymbol{\upsilon}}\newcommand{\biphi}{\boldsymbol{\phi}}\newcommand{\bichi}{\boldsymbol{\chi}}\newcommand{\bipsi}{\boldsymbol{\psi}}\newcommand{\biomega}{\boldsymbol{\omega}}\eta \rm{_p^2}$ = 0.10, but not when the reference moved obliquely, *F*(2.83, 81.95) = 1.65, *p* = 0.19, $\newcommand{\bialpha}{\boldsymbol{\alpha}}\newcommand{\bibeta}{\boldsymbol{\beta}}\newcommand{\bigamma}{\boldsymbol{\gamma}}\newcommand{\bidelta}{\boldsymbol{\delta}}\newcommand{\bivarepsilon}{\boldsymbol{\varepsilon}}\newcommand{\bizeta}{\boldsymbol{\zeta}}\newcommand{\bieta}{\boldsymbol{\eta}}\newcommand{\bitheta}{\boldsymbol{\theta}}\newcommand{\biiota}{\boldsymbol{\iota}}\newcommand{\bikappa}{\boldsymbol{\kappa}}\newcommand{\bilambda}{\boldsymbol{\lambda}}\newcommand{\bimu}{\boldsymbol{\mu}}\newcommand{\binu}{\boldsymbol{\nu}}\newcommand{\bixi}{\boldsymbol{\xi}}\newcommand{\biomicron}{\boldsymbol{\micron}}\newcommand{\bipi}{\boldsymbol{\pi}}\newcommand{\birho}{\boldsymbol{\rho}}\newcommand{\bisigma}{\boldsymbol{\sigma}}\newcommand{\bitau}{\boldsymbol{\tau}}\newcommand{\biupsilon}{\boldsymbol{\upsilon}}\newcommand{\biphi}{\boldsymbol{\phi}}\newcommand{\bichi}{\boldsymbol{\chi}}\newcommand{\bipsi}{\boldsymbol{\psi}}\newcommand{\biomega}{\boldsymbol{\omega}}\eta \rm{_p^2}$ = 0.05. For the upwards reference direction, lower Weber fractions were obtained in the +0° direction difference condition (*M =* 0.29, *SE* = 0.02) than the +90° [*M* = 0.35, *SE* = 0.03, *F*(1, 29) = 5.33, *p* = 0.03, $\newcommand{\bialpha}{\boldsymbol{\alpha}}\newcommand{\bibeta}{\boldsymbol{\beta}}\newcommand{\bigamma}{\boldsymbol{\gamma}}\newcommand{\bidelta}{\boldsymbol{\delta}}\newcommand{\bivarepsilon}{\boldsymbol{\varepsilon}}\newcommand{\bizeta}{\boldsymbol{\zeta}}\newcommand{\bieta}{\boldsymbol{\eta}}\newcommand{\bitheta}{\boldsymbol{\theta}}\newcommand{\biiota}{\boldsymbol{\iota}}\newcommand{\bikappa}{\boldsymbol{\kappa}}\newcommand{\bilambda}{\boldsymbol{\lambda}}\newcommand{\bimu}{\boldsymbol{\mu}}\newcommand{\binu}{\boldsymbol{\nu}}\newcommand{\bixi}{\boldsymbol{\xi}}\newcommand{\biomicron}{\boldsymbol{\micron}}\newcommand{\bipi}{\boldsymbol{\pi}}\newcommand{\birho}{\boldsymbol{\rho}}\newcommand{\bisigma}{\boldsymbol{\sigma}}\newcommand{\bitau}{\boldsymbol{\tau}}\newcommand{\biupsilon}{\boldsymbol{\upsilon}}\newcommand{\biphi}{\boldsymbol{\phi}}\newcommand{\bichi}{\boldsymbol{\chi}}\newcommand{\bipsi}{\boldsymbol{\psi}}\newcommand{\biomega}{\boldsymbol{\omega}}\eta \rm{_p^2}$ = 0.16]; −90° [*M* = 0.36, *SE* = 0.03, *F*(1, 29) = 16.19, *p* < 0.001, $\newcommand{\bialpha}{\boldsymbol{\alpha}}\newcommand{\bibeta}{\boldsymbol{\beta}}\newcommand{\bigamma}{\boldsymbol{\gamma}}\newcommand{\bidelta}{\boldsymbol{\delta}}\newcommand{\bivarepsilon}{\boldsymbol{\varepsilon}}\newcommand{\bizeta}{\boldsymbol{\zeta}}\newcommand{\bieta}{\boldsymbol{\eta}}\newcommand{\bitheta}{\boldsymbol{\theta}}\newcommand{\biiota}{\boldsymbol{\iota}}\newcommand{\bikappa}{\boldsymbol{\kappa}}\newcommand{\bilambda}{\boldsymbol{\lambda}}\newcommand{\bimu}{\boldsymbol{\mu}}\newcommand{\binu}{\boldsymbol{\nu}}\newcommand{\bixi}{\boldsymbol{\xi}}\newcommand{\biomicron}{\boldsymbol{\micron}}\newcommand{\bipi}{\boldsymbol{\pi}}\newcommand{\birho}{\boldsymbol{\rho}}\newcommand{\bisigma}{\boldsymbol{\sigma}}\newcommand{\bitau}{\boldsymbol{\tau}}\newcommand{\biupsilon}{\boldsymbol{\upsilon}}\newcommand{\biphi}{\boldsymbol{\phi}}\newcommand{\bichi}{\boldsymbol{\chi}}\newcommand{\bipsi}{\boldsymbol{\psi}}\newcommand{\biomega}{\boldsymbol{\omega}}\eta \rm{_p^2}$ = 0.36]; and +180° direction difference conditions [*M* = 0.35, *SE* = 0.03, *F*(1, 29) = 8.03, *p* = 0.008, $\newcommand{\bialpha}{\boldsymbol{\alpha}}\newcommand{\bibeta}{\boldsymbol{\beta}}\newcommand{\bigamma}{\boldsymbol{\gamma}}\newcommand{\bidelta}{\boldsymbol{\delta}}\newcommand{\bivarepsilon}{\boldsymbol{\varepsilon}}\newcommand{\bizeta}{\boldsymbol{\zeta}}\newcommand{\bieta}{\boldsymbol{\eta}}\newcommand{\bitheta}{\boldsymbol{\theta}}\newcommand{\biiota}{\boldsymbol{\iota}}\newcommand{\bikappa}{\boldsymbol{\kappa}}\newcommand{\bilambda}{\boldsymbol{\lambda}}\newcommand{\bimu}{\boldsymbol{\mu}}\newcommand{\binu}{\boldsymbol{\nu}}\newcommand{\bixi}{\boldsymbol{\xi}}\newcommand{\biomicron}{\boldsymbol{\micron}}\newcommand{\bipi}{\boldsymbol{\pi}}\newcommand{\birho}{\boldsymbol{\rho}}\newcommand{\bisigma}{\boldsymbol{\sigma}}\newcommand{\bitau}{\boldsymbol{\tau}}\newcommand{\biupsilon}{\boldsymbol{\upsilon}}\newcommand{\biphi}{\boldsymbol{\phi}}\newcommand{\bichi}{\boldsymbol{\chi}}\newcommand{\bipsi}{\boldsymbol{\psi}}\newcommand{\biomega}{\boldsymbol{\omega}}\eta \rm{_p^2}$ = 0.22]; but not the +45° direction difference condition [*M* = 0.30, *SE* = 0.03, *F*(1, 29) = 0.02, *p* = 0.90, $\newcommand{\bialpha}{\boldsymbol{\alpha}}\newcommand{\bibeta}{\boldsymbol{\beta}}\newcommand{\bigamma}{\boldsymbol{\gamma}}\newcommand{\bidelta}{\boldsymbol{\delta}}\newcommand{\bivarepsilon}{\boldsymbol{\varepsilon}}\newcommand{\bizeta}{\boldsymbol{\zeta}}\newcommand{\bieta}{\boldsymbol{\eta}}\newcommand{\bitheta}{\boldsymbol{\theta}}\newcommand{\biiota}{\boldsymbol{\iota}}\newcommand{\bikappa}{\boldsymbol{\kappa}}\newcommand{\bilambda}{\boldsymbol{\lambda}}\newcommand{\bimu}{\boldsymbol{\mu}}\newcommand{\binu}{\boldsymbol{\nu}}\newcommand{\bixi}{\boldsymbol{\xi}}\newcommand{\biomicron}{\boldsymbol{\micron}}\newcommand{\bipi}{\boldsymbol{\pi}}\newcommand{\birho}{\boldsymbol{\rho}}\newcommand{\bisigma}{\boldsymbol{\sigma}}\newcommand{\bitau}{\boldsymbol{\tau}}\newcommand{\biupsilon}{\boldsymbol{\upsilon}}\newcommand{\biphi}{\boldsymbol{\phi}}\newcommand{\bichi}{\boldsymbol{\chi}}\newcommand{\bipsi}{\boldsymbol{\psi}}\newcommand{\biomega}{\boldsymbol{\omega}}\eta \rm{_p^2}$ < 0.01]. This pattern of results suggests that the extent of angular separation between the directions being compared is important for speed discrimination sensitivity. Note that there are two discrepancies between the results reported here and in the preceding experiments (see Figure 7). First, while elevated Weber fractions were also found in the +90°, −90,° and +180° direction difference conditions in Experiment 2, this effect was not restricted to the upwards reference direction, as found here. Second, the reduction in sensitivity when comparing across different directions was not apparent with vertical (upwards/downwards) reference stimuli in Experiment 1.

Next, we aimed to replicate our finding (Experiment 2) of reduced sensitivity to oblique motion compared to cardinal motion. We therefore selected the +0°, +90°, −90° and +180° direction difference conditions and conducted a 2 × 4 ANOVA to assess whether there was an overall effect of reference direction. While there was no significant effect of reference direction, *F*(1, 29) < 0.01, *p* = 0.99, $\newcommand{\bialpha}{\boldsymbol{\alpha}}\newcommand{\bibeta}{\boldsymbol{\beta}}\newcommand{\bigamma}{\boldsymbol{\gamma}}\newcommand{\bidelta}{\boldsymbol{\delta}}\newcommand{\bivarepsilon}{\boldsymbol{\varepsilon}}\newcommand{\bizeta}{\boldsymbol{\zeta}}\newcommand{\bieta}{\boldsymbol{\eta}}\newcommand{\bitheta}{\boldsymbol{\theta}}\newcommand{\biiota}{\boldsymbol{\iota}}\newcommand{\bikappa}{\boldsymbol{\kappa}}\newcommand{\bilambda}{\boldsymbol{\lambda}}\newcommand{\bimu}{\boldsymbol{\mu}}\newcommand{\binu}{\boldsymbol{\nu}}\newcommand{\bixi}{\boldsymbol{\xi}}\newcommand{\biomicron}{\boldsymbol{\micron}}\newcommand{\bipi}{\boldsymbol{\pi}}\newcommand{\birho}{\boldsymbol{\rho}}\newcommand{\bisigma}{\boldsymbol{\sigma}}\newcommand{\bitau}{\boldsymbol{\tau}}\newcommand{\biupsilon}{\boldsymbol{\upsilon}}\newcommand{\biphi}{\boldsymbol{\phi}}\newcommand{\bichi}{\boldsymbol{\chi}}\newcommand{\bipsi}{\boldsymbol{\psi}}\newcommand{\biomega}{\boldsymbol{\omega}}\eta \rm{_p^2}$ < 0.01, and no significant effect of direction difference, *F*(3, 87) = 1.00, *p* = 0.40, $\newcommand{\bialpha}{\boldsymbol{\alpha}}\newcommand{\bibeta}{\boldsymbol{\beta}}\newcommand{\bigamma}{\boldsymbol{\gamma}}\newcommand{\bidelta}{\boldsymbol{\delta}}\newcommand{\bivarepsilon}{\boldsymbol{\varepsilon}}\newcommand{\bizeta}{\boldsymbol{\zeta}}\newcommand{\bieta}{\boldsymbol{\eta}}\newcommand{\bitheta}{\boldsymbol{\theta}}\newcommand{\biiota}{\boldsymbol{\iota}}\newcommand{\bikappa}{\boldsymbol{\kappa}}\newcommand{\bilambda}{\boldsymbol{\lambda}}\newcommand{\bimu}{\boldsymbol{\mu}}\newcommand{\binu}{\boldsymbol{\nu}}\newcommand{\bixi}{\boldsymbol{\xi}}\newcommand{\biomicron}{\boldsymbol{\micron}}\newcommand{\bipi}{\boldsymbol{\pi}}\newcommand{\birho}{\boldsymbol{\rho}}\newcommand{\bisigma}{\boldsymbol{\sigma}}\newcommand{\bitau}{\boldsymbol{\tau}}\newcommand{\biupsilon}{\boldsymbol{\upsilon}}\newcommand{\biphi}{\boldsymbol{\phi}}\newcommand{\bichi}{\boldsymbol{\chi}}\newcommand{\bipsi}{\boldsymbol{\psi}}\newcommand{\biomega}{\boldsymbol{\omega}}\eta \rm{_p^2}$ = 0.03, there was a significant interaction between reference direction and direction difference, *F*(3, 87) = 5.22,* p* = 0.002, $\newcommand{\bialpha}{\boldsymbol{\alpha}}\newcommand{\bibeta}{\boldsymbol{\beta}}\newcommand{\bigamma}{\boldsymbol{\gamma}}\newcommand{\bidelta}{\boldsymbol{\delta}}\newcommand{\bivarepsilon}{\boldsymbol{\varepsilon}}\newcommand{\bizeta}{\boldsymbol{\zeta}}\newcommand{\bieta}{\boldsymbol{\eta}}\newcommand{\bitheta}{\boldsymbol{\theta}}\newcommand{\biiota}{\boldsymbol{\iota}}\newcommand{\bikappa}{\boldsymbol{\kappa}}\newcommand{\bilambda}{\boldsymbol{\lambda}}\newcommand{\bimu}{\boldsymbol{\mu}}\newcommand{\binu}{\boldsymbol{\nu}}\newcommand{\bixi}{\boldsymbol{\xi}}\newcommand{\biomicron}{\boldsymbol{\micron}}\newcommand{\bipi}{\boldsymbol{\pi}}\newcommand{\birho}{\boldsymbol{\rho}}\newcommand{\bisigma}{\boldsymbol{\sigma}}\newcommand{\bitau}{\boldsymbol{\tau}}\newcommand{\biupsilon}{\boldsymbol{\upsilon}}\newcommand{\biphi}{\boldsymbol{\phi}}\newcommand{\bichi}{\boldsymbol{\chi}}\newcommand{\bipsi}{\boldsymbol{\psi}}\newcommand{\biomega}{\boldsymbol{\omega}}\eta \rm{_p^2}$ = 0.15. To understand the source of this interaction, we conducted paired samples *t* tests between the +0°, +90°, −90°, and +180° direction difference conditions in each reference direction. Weber fractions were higher in the +0° direction difference condition with an oblique reference (*M* = 0.35, *SE* = 0.03) than with an upward reference (*M* = 0.29, *SE* = 0.02), and this difference was significant, *t*(29) = 3.39, *p* = 0.002, in line with an oblique effect. However, this oblique effect was not found in the other stimulus conditions. Specifically, in the −90° direction difference condition, slightly lower Weber fractions were found with an oblique reference (*M* = 0.32, *SE* = 0.14) compared to the upward reference (*M* = 0.35, *SE* = 0.16), *t*(29) = 2.07, *p* = 0.05, and the −90° and +180° direction difference conditions did not show significant differences between the two reference directions (*p* ≥ 0.14).

Finally, we assessed direction-related biases in perception. A 5 × 2 ANOVA on PSE values showed a nonsignificant effect of reference direction, *F*(1, 29) = 2.48, *p* = 0.13, $\newcommand{\bialpha}{\boldsymbol{\alpha}}\newcommand{\bibeta}{\boldsymbol{\beta}}\newcommand{\bigamma}{\boldsymbol{\gamma}}\newcommand{\bidelta}{\boldsymbol{\delta}}\newcommand{\bivarepsilon}{\boldsymbol{\varepsilon}}\newcommand{\bizeta}{\boldsymbol{\zeta}}\newcommand{\bieta}{\boldsymbol{\eta}}\newcommand{\bitheta}{\boldsymbol{\theta}}\newcommand{\biiota}{\boldsymbol{\iota}}\newcommand{\bikappa}{\boldsymbol{\kappa}}\newcommand{\bilambda}{\boldsymbol{\lambda}}\newcommand{\bimu}{\boldsymbol{\mu}}\newcommand{\binu}{\boldsymbol{\nu}}\newcommand{\bixi}{\boldsymbol{\xi}}\newcommand{\biomicron}{\boldsymbol{\micron}}\newcommand{\bipi}{\boldsymbol{\pi}}\newcommand{\birho}{\boldsymbol{\rho}}\newcommand{\bisigma}{\boldsymbol{\sigma}}\newcommand{\bitau}{\boldsymbol{\tau}}\newcommand{\biupsilon}{\boldsymbol{\upsilon}}\newcommand{\biphi}{\boldsymbol{\phi}}\newcommand{\bichi}{\boldsymbol{\chi}}\newcommand{\bipsi}{\boldsymbol{\psi}}\newcommand{\biomega}{\boldsymbol{\omega}}\eta \rm{_p^2}$ = 0.08, and a significant effect of direction difference, *F*(4, 116) = 3.74, *p* = 0.007, $\newcommand{\bialpha}{\boldsymbol{\alpha}}\newcommand{\bibeta}{\boldsymbol{\beta}}\newcommand{\bigamma}{\boldsymbol{\gamma}}\newcommand{\bidelta}{\boldsymbol{\delta}}\newcommand{\bivarepsilon}{\boldsymbol{\varepsilon}}\newcommand{\bizeta}{\boldsymbol{\zeta}}\newcommand{\bieta}{\boldsymbol{\eta}}\newcommand{\bitheta}{\boldsymbol{\theta}}\newcommand{\biiota}{\boldsymbol{\iota}}\newcommand{\bikappa}{\boldsymbol{\kappa}}\newcommand{\bilambda}{\boldsymbol{\lambda}}\newcommand{\bimu}{\boldsymbol{\mu}}\newcommand{\binu}{\boldsymbol{\nu}}\newcommand{\bixi}{\boldsymbol{\xi}}\newcommand{\biomicron}{\boldsymbol{\micron}}\newcommand{\bipi}{\boldsymbol{\pi}}\newcommand{\birho}{\boldsymbol{\rho}}\newcommand{\bisigma}{\boldsymbol{\sigma}}\newcommand{\bitau}{\boldsymbol{\tau}}\newcommand{\biupsilon}{\boldsymbol{\upsilon}}\newcommand{\biphi}{\boldsymbol{\phi}}\newcommand{\bichi}{\boldsymbol{\chi}}\newcommand{\bipsi}{\boldsymbol{\psi}}\newcommand{\biomega}{\boldsymbol{\omega}}\eta \rm{_p^2}$ = 0.11. However, we were most interested in the interaction between reference direction and direction difference, which was significant, *F*(2.32, 67.38) = 8.85, *p* < 0.001, $\newcommand{\bialpha}{\boldsymbol{\alpha}}\newcommand{\bibeta}{\boldsymbol{\beta}}\newcommand{\bigamma}{\boldsymbol{\gamma}}\newcommand{\bidelta}{\boldsymbol{\delta}}\newcommand{\bivarepsilon}{\boldsymbol{\varepsilon}}\newcommand{\bizeta}{\boldsymbol{\zeta}}\newcommand{\bieta}{\boldsymbol{\eta}}\newcommand{\bitheta}{\boldsymbol{\theta}}\newcommand{\biiota}{\boldsymbol{\iota}}\newcommand{\bikappa}{\boldsymbol{\kappa}}\newcommand{\bilambda}{\boldsymbol{\lambda}}\newcommand{\bimu}{\boldsymbol{\mu}}\newcommand{\binu}{\boldsymbol{\nu}}\newcommand{\bixi}{\boldsymbol{\xi}}\newcommand{\biomicron}{\boldsymbol{\micron}}\newcommand{\bipi}{\boldsymbol{\pi}}\newcommand{\birho}{\boldsymbol{\rho}}\newcommand{\bisigma}{\boldsymbol{\sigma}}\newcommand{\bitau}{\boldsymbol{\tau}}\newcommand{\biupsilon}{\boldsymbol{\upsilon}}\newcommand{\biphi}{\boldsymbol{\phi}}\newcommand{\bichi}{\boldsymbol{\chi}}\newcommand{\bipsi}{\boldsymbol{\psi}}\newcommand{\biomega}{\boldsymbol{\omega}}\eta \rm{_p^2}$ = 0.23. Given that we expected biases to vary depending on the reference direction, we conducted separate ANOVAs with each reference direction to investigate these biases further. As in Experiment 3, we found that the effect of direction difference was not significant for the upwards reference direction, *F*(2.79, 81.00) = 2.27, *p* = 0.09, $\newcommand{\bialpha}{\boldsymbol{\alpha}}\newcommand{\bibeta}{\boldsymbol{\beta}}\newcommand{\bigamma}{\boldsymbol{\gamma}}\newcommand{\bidelta}{\boldsymbol{\delta}}\newcommand{\bivarepsilon}{\boldsymbol{\varepsilon}}\newcommand{\bizeta}{\boldsymbol{\zeta}}\newcommand{\bieta}{\boldsymbol{\eta}}\newcommand{\bitheta}{\boldsymbol{\theta}}\newcommand{\biiota}{\boldsymbol{\iota}}\newcommand{\bikappa}{\boldsymbol{\kappa}}\newcommand{\bilambda}{\boldsymbol{\lambda}}\newcommand{\bimu}{\boldsymbol{\mu}}\newcommand{\binu}{\boldsymbol{\nu}}\newcommand{\bixi}{\boldsymbol{\xi}}\newcommand{\biomicron}{\boldsymbol{\micron}}\newcommand{\bipi}{\boldsymbol{\pi}}\newcommand{\birho}{\boldsymbol{\rho}}\newcommand{\bisigma}{\boldsymbol{\sigma}}\newcommand{\bitau}{\boldsymbol{\tau}}\newcommand{\biupsilon}{\boldsymbol{\upsilon}}\newcommand{\biphi}{\boldsymbol{\phi}}\newcommand{\bichi}{\boldsymbol{\chi}}\newcommand{\bipsi}{\boldsymbol{\psi}}\newcommand{\biomega}{\boldsymbol{\omega}}\eta \rm{_p^2}$ = 0.07, but was significant for the oblique reference direction, *F*(2.96, 85.87) = 13.40, *p* < 0.001, $\newcommand{\bialpha}{\boldsymbol{\alpha}}\newcommand{\bibeta}{\boldsymbol{\beta}}\newcommand{\bigamma}{\boldsymbol{\gamma}}\newcommand{\bidelta}{\boldsymbol{\delta}}\newcommand{\bivarepsilon}{\boldsymbol{\varepsilon}}\newcommand{\bizeta}{\boldsymbol{\zeta}}\newcommand{\bieta}{\boldsymbol{\eta}}\newcommand{\bitheta}{\boldsymbol{\theta}}\newcommand{\biiota}{\boldsymbol{\iota}}\newcommand{\bikappa}{\boldsymbol{\kappa}}\newcommand{\bilambda}{\boldsymbol{\lambda}}\newcommand{\bimu}{\boldsymbol{\mu}}\newcommand{\binu}{\boldsymbol{\nu}}\newcommand{\bixi}{\boldsymbol{\xi}}\newcommand{\biomicron}{\boldsymbol{\micron}}\newcommand{\bipi}{\boldsymbol{\pi}}\newcommand{\birho}{\boldsymbol{\rho}}\newcommand{\bisigma}{\boldsymbol{\sigma}}\newcommand{\bitau}{\boldsymbol{\tau}}\newcommand{\biupsilon}{\boldsymbol{\upsilon}}\newcommand{\biphi}{\boldsymbol{\phi}}\newcommand{\bichi}{\boldsymbol{\chi}}\newcommand{\bipsi}{\boldsymbol{\psi}}\newcommand{\biomega}{\boldsymbol{\omega}}\eta \rm{_p^2}$ = 0.32. Planned contrasts showed that there were significantly lower PSE values in the +0° direction difference condition (*M* = 6.08, *SE* = 0.05) than the +90°, −90°, and +180° direction difference conditions; +90: *M* = 5.59, *SE* = 0.09, *F*(1, 29) = 25.70, *p* < 0.001, $\newcommand{\bialpha}{\boldsymbol{\alpha}}\newcommand{\bibeta}{\boldsymbol{\beta}}\newcommand{\bigamma}{\boldsymbol{\gamma}}\newcommand{\bidelta}{\boldsymbol{\delta}}\newcommand{\bivarepsilon}{\boldsymbol{\varepsilon}}\newcommand{\bizeta}{\boldsymbol{\zeta}}\newcommand{\bieta}{\boldsymbol{\eta}}\newcommand{\bitheta}{\boldsymbol{\theta}}\newcommand{\biiota}{\boldsymbol{\iota}}\newcommand{\bikappa}{\boldsymbol{\kappa}}\newcommand{\bilambda}{\boldsymbol{\lambda}}\newcommand{\bimu}{\boldsymbol{\mu}}\newcommand{\binu}{\boldsymbol{\nu}}\newcommand{\bixi}{\boldsymbol{\xi}}\newcommand{\biomicron}{\boldsymbol{\micron}}\newcommand{\bipi}{\boldsymbol{\pi}}\newcommand{\birho}{\boldsymbol{\rho}}\newcommand{\bisigma}{\boldsymbol{\sigma}}\newcommand{\bitau}{\boldsymbol{\tau}}\newcommand{\biupsilon}{\boldsymbol{\upsilon}}\newcommand{\biphi}{\boldsymbol{\phi}}\newcommand{\bichi}{\boldsymbol{\chi}}\newcommand{\bipsi}{\boldsymbol{\psi}}\newcommand{\biomega}{\boldsymbol{\omega}}\eta \rm{_p^2}$ = 0.47; −90: *M* = 5.62, *SE* = 0.09, *F*(1, 29) = 22.73, *p* < 0.001, $\newcommand{\bialpha}{\boldsymbol{\alpha}}\newcommand{\bibeta}{\boldsymbol{\beta}}\newcommand{\bigamma}{\boldsymbol{\gamma}}\newcommand{\bidelta}{\boldsymbol{\delta}}\newcommand{\bivarepsilon}{\boldsymbol{\varepsilon}}\newcommand{\bizeta}{\boldsymbol{\zeta}}\newcommand{\bieta}{\boldsymbol{\eta}}\newcommand{\bitheta}{\boldsymbol{\theta}}\newcommand{\biiota}{\boldsymbol{\iota}}\newcommand{\bikappa}{\boldsymbol{\kappa}}\newcommand{\bilambda}{\boldsymbol{\lambda}}\newcommand{\bimu}{\boldsymbol{\mu}}\newcommand{\binu}{\boldsymbol{\nu}}\newcommand{\bixi}{\boldsymbol{\xi}}\newcommand{\biomicron}{\boldsymbol{\micron}}\newcommand{\bipi}{\boldsymbol{\pi}}\newcommand{\birho}{\boldsymbol{\rho}}\newcommand{\bisigma}{\boldsymbol{\sigma}}\newcommand{\bitau}{\boldsymbol{\tau}}\newcommand{\biupsilon}{\boldsymbol{\upsilon}}\newcommand{\biphi}{\boldsymbol{\phi}}\newcommand{\bichi}{\boldsymbol{\chi}}\newcommand{\bipsi}{\boldsymbol{\psi}}\newcommand{\biomega}{\boldsymbol{\omega}}\eta \rm{_p^2}$ = 0.44; +180°: *M* = 5.69, *SE* = 0.09, *F*(1, 29) = 12.55, *p* = 0.001, $\newcommand{\bialpha}{\boldsymbol{\alpha}}\newcommand{\bibeta}{\boldsymbol{\beta}}\newcommand{\bigamma}{\boldsymbol{\gamma}}\newcommand{\bidelta}{\boldsymbol{\delta}}\newcommand{\bivarepsilon}{\boldsymbol{\varepsilon}}\newcommand{\bizeta}{\boldsymbol{\zeta}}\newcommand{\bieta}{\boldsymbol{\eta}}\newcommand{\bitheta}{\boldsymbol{\theta}}\newcommand{\biiota}{\boldsymbol{\iota}}\newcommand{\bikappa}{\boldsymbol{\kappa}}\newcommand{\bilambda}{\boldsymbol{\lambda}}\newcommand{\bimu}{\boldsymbol{\mu}}\newcommand{\binu}{\boldsymbol{\nu}}\newcommand{\bixi}{\boldsymbol{\xi}}\newcommand{\biomicron}{\boldsymbol{\micron}}\newcommand{\bipi}{\boldsymbol{\pi}}\newcommand{\birho}{\boldsymbol{\rho}}\newcommand{\bisigma}{\boldsymbol{\sigma}}\newcommand{\bitau}{\boldsymbol{\tau}}\newcommand{\biupsilon}{\boldsymbol{\upsilon}}\newcommand{\biphi}{\boldsymbol{\phi}}\newcommand{\bichi}{\boldsymbol{\chi}}\newcommand{\bipsi}{\boldsymbol{\psi}}\newcommand{\biomega}{\boldsymbol{\omega}}\eta \rm{_p^2}$ = 0.30, with an oblique reference direction. Meanwhile, the +45° direction difference condition (*M* = 6.37, *SE* = 0.15) did not differ significantly from the +0° direction difference condition, *F*(1, 29) = 3.78, *p* = 0.06, $\newcommand{\bialpha}{\boldsymbol{\alpha}}\newcommand{\bibeta}{\boldsymbol{\beta}}\newcommand{\bigamma}{\boldsymbol{\gamma}}\newcommand{\bidelta}{\boldsymbol{\delta}}\newcommand{\bivarepsilon}{\boldsymbol{\varepsilon}}\newcommand{\bizeta}{\boldsymbol{\zeta}}\newcommand{\bieta}{\boldsymbol{\eta}}\newcommand{\bitheta}{\boldsymbol{\theta}}\newcommand{\biiota}{\boldsymbol{\iota}}\newcommand{\bikappa}{\boldsymbol{\kappa}}\newcommand{\bilambda}{\boldsymbol{\lambda}}\newcommand{\bimu}{\boldsymbol{\mu}}\newcommand{\binu}{\boldsymbol{\nu}}\newcommand{\bixi}{\boldsymbol{\xi}}\newcommand{\biomicron}{\boldsymbol{\micron}}\newcommand{\bipi}{\boldsymbol{\pi}}\newcommand{\birho}{\boldsymbol{\rho}}\newcommand{\bisigma}{\boldsymbol{\sigma}}\newcommand{\bitau}{\boldsymbol{\tau}}\newcommand{\biupsilon}{\boldsymbol{\upsilon}}\newcommand{\biphi}{\boldsymbol{\phi}}\newcommand{\bichi}{\boldsymbol{\chi}}\newcommand{\bipsi}{\boldsymbol{\psi}}\newcommand{\biomega}{\boldsymbol{\omega}}\eta \rm{_p^2}$ = 0.12. Corroborating this pattern of results, one-sample *t* tests showed that the +90°, −90°, and +180° direction difference conditions for the oblique reference direction all led to significantly lower PSE values than 6°/s; +90°: *t*(29) = 4.76, *p* < 0.001; −90°: *t*(29) = 4.46, *p* < 0.001; +180°: *t*(29) = 3.42, *p* = 0.002, indicating that the oblique comparison stimuli were perceived to be moving faster than the oblique reference stimulus. Conversely, and in line with Experiment 3, the +45° direction difference condition with an oblique reference had a PSE value significantly *above* 6°/s, *t*(29) = 2.55, *p* = 0.02, reflecting that the horizontal comparison stimulus was perceived to be moving more slowly than the oblique reference stimulus. Meanwhile, the PSE value in the +45° direction difference condition with an upwards reference did not differ significantly from 6°/s, *t*(29) = 1.23, *p* = 0.23, suggesting that the oblique stimuli were not perceived as moving faster than vertical stimuli. This result is in line with Experiment 2, but not in Experiment 1, where we reported a bias for perceiving oblique stimuli as moving faster than vertical stimuli.

In summary, the participants in Experiment 4 were less sensitive to speed differences in stimuli moving in different directions compared to those moving in the same direction, but only when the reference moved upwards, as opposed to obliquely (cf. Experiments 1 and 2). With the upwards reference, reduced sensitivity was only found in the +90°, −90°, and +180° direction difference conditions, but not the +45° condition, suggesting that the extent of angular separation between the directions being compared is important for speed discrimination sensitivity. An oblique effect was apparent when comparing across the +0° conditions, but not when comparing sensitivity for other pairs of cardinal or oblique stimuli (cf. Experiment 2). Direction-related biases were found, with the oblique reference stimulus perceived to move faster than the horizontal stimulus (see also Experiment 3), but there was no evidence of this oblique stimulus being perceived to move faster than vertical (cf. Experiment 1). Additionally, there were biases in the +90°, −90°, and +180° direction difference conditions with an oblique reference stimulus, which we were not predicting on the basis of the preceding experiments.

## Pooled data across experiments

Fifty-three participants completed the +0° and +90° direction difference conditions for upwards and oblique reference directions across Experiments 2, 3, and 4, which meant that these data could be pooled to characterize more conclusively oblique effects in sensitivity. A 2 × 2 ANOVA revealed significant main effects of reference direction, *F*(1, 52) = 5.27, *p* = 0.03, $\newcommand{\bialpha}{\boldsymbol{\alpha}}\newcommand{\bibeta}{\boldsymbol{\beta}}\newcommand{\bigamma}{\boldsymbol{\gamma}}\newcommand{\bidelta}{\boldsymbol{\delta}}\newcommand{\bivarepsilon}{\boldsymbol{\varepsilon}}\newcommand{\bizeta}{\boldsymbol{\zeta}}\newcommand{\bieta}{\boldsymbol{\eta}}\newcommand{\bitheta}{\boldsymbol{\theta}}\newcommand{\biiota}{\boldsymbol{\iota}}\newcommand{\bikappa}{\boldsymbol{\kappa}}\newcommand{\bilambda}{\boldsymbol{\lambda}}\newcommand{\bimu}{\boldsymbol{\mu}}\newcommand{\binu}{\boldsymbol{\nu}}\newcommand{\bixi}{\boldsymbol{\xi}}\newcommand{\biomicron}{\boldsymbol{\micron}}\newcommand{\bipi}{\boldsymbol{\pi}}\newcommand{\birho}{\boldsymbol{\rho}}\newcommand{\bisigma}{\boldsymbol{\sigma}}\newcommand{\bitau}{\boldsymbol{\tau}}\newcommand{\biupsilon}{\boldsymbol{\upsilon}}\newcommand{\biphi}{\boldsymbol{\phi}}\newcommand{\bichi}{\boldsymbol{\chi}}\newcommand{\bipsi}{\boldsymbol{\psi}}\newcommand{\biomega}{\boldsymbol{\omega}}\eta \rm{_p^2}$ = 0.09, and direction difference condition, *F*(1, 52) = 5.38, *p* = 0.02, $\newcommand{\bialpha}{\boldsymbol{\alpha}}\newcommand{\bibeta}{\boldsymbol{\beta}}\newcommand{\bigamma}{\boldsymbol{\gamma}}\newcommand{\bidelta}{\boldsymbol{\delta}}\newcommand{\bivarepsilon}{\boldsymbol{\varepsilon}}\newcommand{\bizeta}{\boldsymbol{\zeta}}\newcommand{\bieta}{\boldsymbol{\eta}}\newcommand{\bitheta}{\boldsymbol{\theta}}\newcommand{\biiota}{\boldsymbol{\iota}}\newcommand{\bikappa}{\boldsymbol{\kappa}}\newcommand{\bilambda}{\boldsymbol{\lambda}}\newcommand{\bimu}{\boldsymbol{\mu}}\newcommand{\binu}{\boldsymbol{\nu}}\newcommand{\bixi}{\boldsymbol{\xi}}\newcommand{\biomicron}{\boldsymbol{\micron}}\newcommand{\bipi}{\boldsymbol{\pi}}\newcommand{\birho}{\boldsymbol{\rho}}\newcommand{\bisigma}{\boldsymbol{\sigma}}\newcommand{\bitau}{\boldsymbol{\tau}}\newcommand{\biupsilon}{\boldsymbol{\upsilon}}\newcommand{\biphi}{\boldsymbol{\phi}}\newcommand{\bichi}{\boldsymbol{\chi}}\newcommand{\bipsi}{\boldsymbol{\psi}}\newcommand{\biomega}{\boldsymbol{\omega}}\eta \rm{_p^2}$ = 0.09, and an interaction between reference direction and direction difference condition, *F*(1, 52) = 11.54, *p* = 0.001, $\newcommand{\bialpha}{\boldsymbol{\alpha}}\newcommand{\bibeta}{\boldsymbol{\beta}}\newcommand{\bigamma}{\boldsymbol{\gamma}}\newcommand{\bidelta}{\boldsymbol{\delta}}\newcommand{\bivarepsilon}{\boldsymbol{\varepsilon}}\newcommand{\bizeta}{\boldsymbol{\zeta}}\newcommand{\bieta}{\boldsymbol{\eta}}\newcommand{\bitheta}{\boldsymbol{\theta}}\newcommand{\biiota}{\boldsymbol{\iota}}\newcommand{\bikappa}{\boldsymbol{\kappa}}\newcommand{\bilambda}{\boldsymbol{\lambda}}\newcommand{\bimu}{\boldsymbol{\mu}}\newcommand{\binu}{\boldsymbol{\nu}}\newcommand{\bixi}{\boldsymbol{\xi}}\newcommand{\biomicron}{\boldsymbol{\micron}}\newcommand{\bipi}{\boldsymbol{\pi}}\newcommand{\birho}{\boldsymbol{\rho}}\newcommand{\bisigma}{\boldsymbol{\sigma}}\newcommand{\bitau}{\boldsymbol{\tau}}\newcommand{\biupsilon}{\boldsymbol{\upsilon}}\newcommand{\biphi}{\boldsymbol{\phi}}\newcommand{\bichi}{\boldsymbol{\chi}}\newcommand{\bipsi}{\boldsymbol{\psi}}\newcommand{\biomega}{\boldsymbol{\omega}}\eta \rm{_p^2} =$ 0.18. In line with the conclusions from Experiment 4, this interaction effect was driven by an oblique effect in sensitivity which was present when comparing the upwards and oblique reference directions for the +0° direction difference condition [upwards: *M* = 0.26, *SE* = 0.01; oblique: *M* = 0.31, *SE* = 0.02; *t*(52) = 4.39, *p* < 0.001], but not when comparing across the +90° direction difference condition [upwards: *M* = 0.32, *SE* = 0.02; oblique: *M* = 0.31, *SE* = 0.02; *t*(52) = 0.94, *p* = 0.35]. Further corroborating our findings from Experiment 4, participants were poorer at judging speeds moving in directions separated by +90° than stimuli moving in the same direction only when the reference was upwards, *t*(52) = 3.81, *p* < 0.001, and not oblique, *t*(52) = 0.23, *p* = 0.82.

## General discussion

In a series of experiments, we investigated whether speed discrimination is hindered when comparing across motions in different directions, while additionally looking for the existence of an oblique effect and direction-related biases in speed perception. Our results suggest that both absolute and relative direction affect speed discrimination sensitivity, and that perceived speed depends on direction.

### 

#### Sensitivity across directions

The main question we sought to address was whether speed discrimination sensitivity was reduced when comparing across two different motion directions than when comparing across stimuli moving in the same direction. Overall, our results suggested that this was the case, although the effect was modest and not entirely consistent across our experiments. We found evidence for reduced sensitivity for comparing across directions in twelve participants in Experiment 2, but not in Experiments 1 and 3, and in the larger sample (*n* = 30) in Experiment 4, we found that the cost of comparing across different directions was restricted to stimuli with an upwards reference as opposed to an oblique reference. The reason for this interaction between absolute direction (reference direction) and relative direction (direction of reference relative to comparison) is not clear, but it could be because the upwards reference stimulus is more precisely represented than the oblique reference stimulus (i.e., an “oblique effect,” as we discuss below), meaning that the effect of comparing across directions is more apparent in this case.

The effects of relative direction on speed discrimination suggest that speed and direction are not processed wholly independently. Perhaps this conclusion is unsurprising, given that speed discrimination is presumed to depend on signals in directionally selective neurons (Maunsell & Van Essen, 1983; Mikami et al., 1986). Adaptation studies have also suggested that speed and direction are not processed independently (Schrater & Simoncelli, 1998; Stocker & Simoncelli, 2009). Yet, there are also many reports of dissociations between speed and direction discrimination (e.g., Matthews & Qian, 1999; Saffell & Matthews, 2003; see Nishida, 2011, for review). In the context of this previous work we conclude that while speed discrimination may draw on some distinct neural resources for direction discrimination, it does not depend on processes that are wholly direction independent. This conclusion is consistent with the proposal by Stocker and Simoncelli (2009) that both directional and nondirectional mechanisms contribute to velocity discrimination. Additionally, we note that performance in another discrimination task (trajectory orientation discrimination) is poorer when comparing stimuli moving in different directions than those moving in the same direction (Matthews & Allen, 2005). Notably, however, our conclusion contrasts that of Burbeck and Regan (1983) who investigated comparisons of spatial frequency for different orientations (and vice versa), and concluded that these stimulus dimensions are processed independently.

The precision with which comparison can be made between neural signals depends, of course, on the level of noise within the signals to be compared. While correlated noise between similar neurons reduces the benefits of pooling their responses, it does not diminish the ability to gain information from comparing their responses, e.g., to judge direction or speed (Zohary, Shadlen, & Newsome, 1994; Kohn, Coen-Cagli, Kanitscheider, & Pouget, 2016). However, if the correlation is stronger between neurons with the same stimulus tuning than between those with different tuning, then the ability to gain information about the stimulus dimension is diminished (Kohn et al., 2016). Zohary et al. (1994) found that for directionally tuned neurons in macaque area MT, the noise correlation is stronger between nearby neurons having a similar directional tuning than between those whose preferred directions differ by 90° or more. Thus, if speed comparison between different directions depends on comparing signals from these latter different neuronal pools, this provides a possible reason why such discrimination is worse between different motions than between motions in the same direction. There may also be differences in the connections between sensory inputs and the decision-stage in the two cases (Petrov, Dosher, & Lu, 2005).

Our stimulus presentation required observers to not only compare speed signals across different directions, but also across different spatial locations. Therefore performance in our task may be further constrained by the properties of the high-level mechanism that compares motion signals across space (Maruya, Holcombe, & Nishida, 2013). To examine the properties of such a comparison mechanism in the context of speed discrimination, future studies could investigate the effects of manipulating the spatial separation and layout of stimuli on speed discrimination performance. We note, however, that people are still relatively good at comparing speed information even when the motions to be compared are in different directions and different spatial locations—leaving open the broader question of how observers compare representations carried by different populations of neurons (Danilova & Mollon, 2003).

#### Oblique effect

In addition to our main question on the efficacy of discriminating speeds across different directions, we also collected extensive data testing the existence of an oblique effect in speed discrimination. Although it has been claimed that the oblique effect is found only for direction discrimination and not speed discrimination (Matthews & Qian, 1999; Westheimer, 2003), we found that observers were poorer at discriminating between two stimuli moving in the same, oblique direction compared to two stimuli moving in the same, upwards direction. Perhaps this discrepancy with previous studies arises from differences in stimuli, procedure, and sample size. Specifically, Matthews and Qian (1999) and Westheimer (2003) presented sequentially presented stimuli, compared to the simultaneously presented stimuli used here, and used smaller groups of participants (*n =* 12 and *n* = 3, respectively). Indeed, it is possible that the oblique effect for speed discrimination is of a much smaller magnitude than that shown for direction discrimination (Matthews & Qian, 1999).

It should be noted that we draw our conclusions about oblique effects from the case where the motions to be compared are in the same (either cardinal or oblique) direction. This is in line with previous investigations of oblique effects in speed discrimination (Matthews & Qian, 1999; Westheimer 2003) which have similarly been based on pairs of stimuli moving in the same direction. Thus the general impact of the oblique effect found here is currently unclear. In Experiment 2, we found that observers were generally poorer at speed discrimination for all pairs of oblique stimuli as opposed to pairs of cardinal stimuli, whereas the oblique effect was only apparent when comparing across stimuli moving in the same direction as each other in Experiment 4 and in the pooled analysis across Experiments 2, 3, and 4. As we only compared one oblique reference stimulus with one cardinal reference stimulus (upwards), the oblique effect reported here will need to be extended to other cardinal and oblique directions. Yet, the existence of anisotropies in speed discrimination will be important in refining models of oblique effects in direction perception (e.g., Rokem & Silver, 2009; Wong & Price, 2014). If an oblique effect for direction is proposed to arise from increased representation of cardinal versus oblique motions in the visual cortex (Furmanski & Engel, 2000; Li, Peterson, & Freeman, 2003) and/or in the statistics of the environment (Dakin et al., 2005), it might be expected that these factors should also influence speed discrimination.

#### Direction-dependent biases

Our results also revealed direction-dependent biases on speed judgments, whereby oblique stimuli appeared to move faster than horizontal stimuli in Experiments 3 and 4. Whereas Experiment 1 suggested that oblique stimuli were also perceived to move faster than vertical stimuli, this finding was not replicated in Experiment 3 or the larger sample in Experiment 4. Perhaps this bias was not apparent in Experiments 3 and 4 because the oblique (+45°) stimulus direction was also the reference direction for half of the trials in these experiments, unlike in Experiment 1. Surprisingly, in Experiment 4, we also found that the oblique reference stimulus was perceived to be moving more slowly than comparison stimuli moving in other oblique directions. The reason for these biases will need further investigation. It is possible that the repetition of vertical and oblique directions in the reference stimuli may have led to adaptation inducing shifts in perceived speed. We also note that there may be important relationships between bias and sensitivity. For example, in Experiment 4, significant biases were found for the oblique reference direction but not the upwards reference direction, whereas the reverse pattern was found for Weber fractions – with effects of condition only for the upwards reference direction and not the oblique reference direction. Perhaps, therefore, the increased sensitivity to the upwards reference stimulus compared to the oblique reference stimulus made it comparatively resistant to biased perception. It is also possible that biases could arise at the decision stage, rather than the perceptual stage (Morgan, Melmoth, & Solomon, 2013).

#### Individual differences

As we alluded to in the preceding discussion, there were some inconsistencies in the results of the four experiments. It is possible that these differences emerged due to the unique set of conditions presented in each experiment, leading to certain directions being more or less represented than others. However, it is also likely that the difficulty in replicating effects in our smaller groups (Experiments 1 through 3) arises from considerable between-participant variability, as reflected in the wide distribution of individual Weber fractions and PSE values in Figures 3 through 6. Considerable individual differences in perception, including motion perception, have been reported previously (Bosten et al., 2017; Halpern et al., 1999), and we propose that these individual differences may extend to the pattern of differential sensitivity reported here. Yet, to clearly separate true individual differences from measurement error, it will be necessary to evaluate the test-retest reliability of our psychophysical estimates. However, we note that the data were well fitted by psychometric functions and that we excluded inattentive participants who made frequent mistakes on catch-trials. We propose, therefore, that the considerable individual differences in psychophysical estimates reflect more than just measurement error. Such individual differences could also help explain previous discrepant results, including those between Verghese and McKee (2006) and Manning et al. (2015). Generally, studies of speed discrimination may require larger participant samples than the common practice in visual psychophysics.

Rather than reflecting merely “noise,” the existence of individual differences could potentially inform on important differences in the mechanisms underlying speed judgments (Mollon, Bosten, Peterzell, & Webster, 2017). Yet more work is needed to understand why individuals differ, for example by investigating correlations between individual differences in this task and those in other tasks, or those in neurophysiological measures. The investigation of individual differences in motion perception has already proven fruitful for understanding vision in children (Braddick et al., 2016, 2017). Moreover, atypical motion processing has been implicated in a range of neurodevelopmental and neuropsychiatric disorders (Braddick, Atkinson, & Wattam-Bell, 2003; Chen, 2011; Atkinson, 2017; Porter et al., 2017), and the results reported here have lessons for studies that depend on speed and/or direction discrimination.

## Conclusions

We conclude that speed judgments depend on both absolute and relative direction, which appear to interact with each other. Although there was considerable variability between participants, we report evidence of (a) reduced sensitivity for stimuli moving in different directions, (b) an oblique effect in speed discrimination when comparing pairs of stimuli moving in the same direction, and (c) direction-dependent biases. These results suggest that speed and direction information cannot be processed entirely independently, thus informing our understanding of how information is extracted from their joint representation.
